# Metastatic organotropism in peritoneal metastasis: Paget’s hypothesis revisited

**DOI:** 10.1007/s10238-025-01868-9

**Published:** 2026-01-29

**Authors:** Dongchan Kim, Devesh Kaushal, Robert Beaumont Wilson

**Affiliations:** 1 Orange Health Service, Orange, NSW 2800 Australia; 2 Department of General Surgery, Campbelltown Hospital, Sydney, NSW 2560 Australia; 3 Faculty of Medicine, Liverpool Clinical School, University of New South Wales, Sydney, NSW 2170 Australia

**Keywords:** Exosomes, Extracellular vesicles, Peritoneal metastasis, PM, Paget’s theory

## Abstract

Peritoneal metastasis (PM) of solid tumours is a major contributor to cancer-associated mortality and morbidity. The mechanism of PM development encapsulates Paget’s hypothesis of seed and soil, whereby cancer cells remotely prepare a pre-metastatic niche in the peritoneal microenvironment to facilitate transcoelomic cancer progression. The bidirectional communication between cancer cells and host mesothelial cells, endothelial cells, leukocytes, adipocytes, and fibroblasts occurs via exosomes. Exosomes are nano-sized extracellular vesicles that carry cargos of proteins, cytokines, and microRNA. Cancer-derived exosomes enable exfoliated tumour cells to resist anoikis, disseminate, adhere, and implant in the peritoneum. This process involves the degradation of the peritoneal glycocalyx, the transformation of peritoneal mesothelial cells into cancer-associated fibroblasts via mesothelial-mesenchymal transition, and metabolic coupling with omental and subperitoneal adipocytes. Exosomes also enhance ascites and peritoneal immunosuppression. Exosomes promote PM development from mesenchymal subtypes of epithelial cancers, which have a predilection for transcoelomic metastasis compared to other molecular subtypes. Mesenchymal subtypes include diffuse gastric cancer, CMS4 colorectal cancer, and high-grade serous ovarian carcinoma. Understanding the oncogenic roles of exosomal cargo offers potential for future research and therapy in PM.

## Introduction

The concept of cross-talk between cancer cells and distant organs was first proposed in 1889 by the English surgeon Stephen Paget in his “seed and soil” hypothesis [1]. Using post-mortem findings from 735 patients with fatal breast cancer, Paget’s seminal work reported on the selective, organotropic patterns of metastatic dissemination to the liver, lungs, cranium and medulla of long bones [1, 2]. Paget suggested that the receptive tissue of distant organs contributed to the specific sites of cancer metastasis [1, 2]. Paget’s hypothesis was in contrast to James Ewing’s 1928 hypothesis, which posited that the embolism of cancer cells within the vasculature, along with the anatomical and mechanical features of venous and lymphatic drainage, dictated the location of metastasis (*anatomical and mechanical hypothesis*) [2, 3]*.* Paget’s hypothesis gained prominence only when animal experiments provided confirmatory evidence of preferential metastasis in the 1960s [4, 5].

In the 1980s, in vitro evidence demonstrated that preferential metastases correlate with the adhesion of circulating tumour cells (CTCs) to the microvascular endothelium [6], including the capillaries of the leptomeninges [7]. Extensive clinical and experimental research has now validated Paget’s hypothesis of organotropic metastasis, which arises as a result of favourable interactions between cancer cells (the “seed”) and receptive microenvironments and vasculature of distant organs (the “soil”) [8, 9]. For instance, the microenvironments of hepatic sinusoids, bone marrow sinusoids, and the pulmonary microvasculature commonly attract metastatic cancer cells due to their incomplete, fenestrated, or receptive endothelium [8]. Such chemotaxis is facilitated by CTC interactions with host neutrophils and exosomes released by cancer cells [10]. The endothelium of these sites has unique characteristics which can provide a *pre-metastatic niche* [11]. Similarly, omental milky spots contain incomplete peritoneal mesothelium with exposed extracellular matrix (ECM) protein ligands [12, 13]. Omental milky spots also contain high endothelial venules (HEVs), which facilitate the constitutive extravasation of neutrophils and lymphocytes, primarily via paracellular diapedesis [13, 14]. Milky spots have a central hypoxic microenvironment, within which hypoxia-inducible factor 1α (HIF-1α) stimulates the formation of the pre-metastatic niche [15]. HEVs in omental milky spots are specialised structures that allow leukocyte extravasation via integrin interactions, which do not require leukocyte selectins (L-selectins) [16].

Exosomes and ascites promote the formation of a pre-metastatic niche in the peritoneum. Exosomal cargo proteins and genetic material facilitate the breakdown of the mesothelial barrier, and induce molecular and cellular changes that promote the development of PM. Exosomal transfer of oncogenic molecules from mesenchymal-type tumours is also a key mechanism for PM development from solid tumours. This review will highlight the biology and contribution of exosomes to PM development, the anatomical and molecular processes underpinning PM formation, and the predilection for PM by mesenchymal-type solid tumours.

## Exosome biology and biogenesis

Exosomes are nano-sized extracellular vesicles enclosed by a lipid bilayer membrane, constitutively produced from the multivesicular bodies (MVBs) of eukaryotic cells [17–20]. Exosomes are found in most bodily fluids, including blood plasma, lymph, saliva, urine, breast milk, sweat, tears and ascites [18]. Exosome membranes contain lipids including ceramides, cholesterol, glycolipids and sphingomyelin; and transmembrane proteins including adhesion molecules (EpCAM, intercellular adhesion molecule (ICAM), integrins αvβ3, αvβ5, α5β1, α6β4, α4β1, αvβ6), molecular chaperones, tetraspanins (CD63, CD81, CD9), and major histocompatibility complex (MHC) molecules [21, 22]. Enveloped within the exosome are nucleotides (DNA, microRNA (miRNA), long non-coding RNA (lncRNA), circular RNA (circRNA), and transfer RNA), proteins, enzymes, growth factors, amino acids, lipids, and metabolites [18, 21]. The composition of the exosomal cargo reflects the parent cell of origin, but this can vary if cells are stressed, stimulated, differentiated, or transformed [17, 18].

With the transfer of cargo contents, exosomes can mediate autocrine, paracrine, and endocrine communication between cells or distant organs via signal transduction, antigen presentation, immune response, and genomic transfer [17, 18]. The delivery of exosomal cargo occurs through endocytosis and internalisation of exosomes by recipient cells [23]. Exosomes can reprogram cellular functions and affect biological and metabolic processes, such as glycolysis, mitochondrial activity, migration and invasion of tumour or mesothelial cells, and angiogenesis and vascular leakage of endothelial cells in the peritoneal cavity [23].

Exosome production begins with endocytosis at the cell membrane, which buds inward to form endosomes [18]. Endosomes mature and enclose proteins, lipids, and nucleic acids, to develop multiple intraluminal vesicles (ILVs) [18]. With maturation, ILVs can fuse and become MVBs [18]. This sequential process of ILV and MVB formation is catalysed by a multiprotein complex termed the ‘endosomal sorting complex required for transport’ (ESCRT) [24]. ESCRT comprises four different complexes, each with a unique role in exosome production. ESCRT-0 clusters ubiquitylated proteins in endosomes [25]. ESCRT-I and -II synergistically promote endocytic membrane budding with clustered cargo [25]. Subsequently, ESCRT-III facilitates vesicle scission [25]. These components of ESCRT operate in a cascade of molecular processes to facilitate the formation of MVBs [24].

## Autophagy, exocytosis and molecular machinery

The MVBs can either fuse with cytoplasmic lysosomes for degradation during autophagy, or be trafficked to and fuse with the plasma membrane for exocytosis as exosomes [26, 27]. Multiple associated proteins help catalyse each process, such as GTPases of the Ras-associated binding protein (Rab) family that promote intracellular trafficking along cytoskeletal actin filaments or microtubules and vesicle fusion with cell membranes [28, 29]. The c-Src kinase phosphorylates proteins that regulate exosome biogenesis and secretion, including syntenin and syndecans, and apoptosis-linked gene-2-interacting protein X (ALIX), an ESCRT-interacting molecule [30, 31]. Src can activate ESCRT-mediated ILV formation [30] and stimulate exosome secretion [31].

Small Rab GTPases regulate the transport of exosomes, as well as constitutive and regulated exocytosis [32, 33]. Rab27 is a Rab GTPase that facilitates exosome secretion [32, 33]. Rab27B is a Rab27 homologue involved in invasive tumour growth, and stimulates G1/S cell cycle transition and cancer cell proliferation [32]. Xenograft mouse models have demonstrated that Rab27B-knockdown reduces in vivo development of PM from gastric cancer cells [33]. Upregulation of Rab27B mRNA in tumour tissue significantly correlates with shorter overall survival and recurrence-free survival in patients with gastric cancer [33]. Rab27B enhances the release of pro-invasive growth regulators from breast cancer cells, such as heat shock protein (HSP)-90α [32].

## Exosome release and invadopodia

Cells can spontaneously secrete exosomes, although extracellular acidosis, a rise in intracellular calcium levels, or expression of calcium-dependent membrane proteins all increase exosome secretion [34]. In OVCAR-3 epithelial ovarian cancer (EOC) cells, the chelation of extracellular calcium triggers the secretion of exosomes [34]. Such chelation-induced exosomes contain a distinct miRNA profile with specific effects on recipient cells, such as increased migration of cancer-associated fibroblasts (CAFs), compared to constitutively secreted exosomes [34]. Upregulation of sirtuin 1 in the tumour microenvironment (TME) can also elicit exosome release from ovarian cancer cells [35]. Hypoxia increases the number of exosomes released by cancer cells due to HIF-1α activating the small GTPase Rab22A and the actin regulator RHO-associated protein kinase (ROCK) [36]. Increased extracellular vesicle shedding is also related to hypoxia-induced cysteine protease calpain expression and activation in macrophages [36].

Exosome secretion from cancer cells holds special significance, as exosome secretion can occur at invadopodia [37]. Invadopodia of cancer cells are finger-like protrusions of the cytoplasm containing actin structures, which are crucial in mediating cancer cell invasion [37]. Invadopodia formation can occur in response to hypoxia, cytosolic glycolysis, and extracellular acidosis [38, 39]. Exosome precursors are transported by the kinesin and dynein motor proteins along actin filaments [40], and localise to invadopodia [37]. Rab GTPases and soluble N-ethylmaleimide-sensitive factor attachment protein receptor (SNARE) proteins promote the fusion of MVBs with the cell membrane, leading to exosome secretion [40]. Cancer cell secretion of exosomes increases with a stiff ECM, which activates Akt and promotes GTP loading to Rab8 [41].

## Suppression of exosome biogenesis and release

Exosomes can be suppressed by inhibiting the biogenesis, intracellular trafficking and exocytosis of MVBs [42]. Incorporation of lipids into the lipid bilayer membrane is required for membrane fluidity, formation of MVBs, and exosome release [43]. Blockade of various targets can inhibit this process:

1. The synthesis of ceramide from sphingomyelin by neutral sphingomyelinase (GW4869, spiroepoxide, 2,6-dimethoxy-4-(5-phenyl-4-thiophen-2-yl-1H-imidazole-2-yl)-phenol (DPTIP)), and acid sphingomyelinase (imipramine) [43]
2. Cholesterol synthesis (simvastatin; HMG-CoA reductase inhibitor) [43]
3. Translocation of phosphatidylserine (D-pantethine; vitamin B5 derivative) [43]
4. Calcium channels (dimethyl amiloride, ketotifen) [43]

Potent farnesyl transferase inhibitors, such as tipifarnib, can suppress exosome biogenesis and inhibit the metastasis of prostate cancer cells [42]. Tipifarnib disrupts Ras/Raf/ERK signalling pathways, and decreases the expression of ALIX, neutral sphingomyelinase 2 (nSMase2), and Rab27A—a GTPase that regulates exosome exocytosis [42, 44]. The RAS inhibitor manumycin A can reduce exosome secretion in prostate cancer cells by nearly 55%, without affecting benign cells [42]. The mammalian target of rapamycin complex 1 (mTORC1) modulates autophagy of late endosomes and also inhibits Rab27A [19, 44]. Rapamycin can inhibit mTORC1, thereby promoting the release of exosomes [44]. Exosome secretion from prostate cancer can also be reduced by calpain inhibitors and ROCK inhibitors, such as calpeptin and Y27632, respectively [42].

Endocytosis of formed exosomes or their future cargo are clathrin-dependent (inhibited by Dynasore) or clathrin-independent (inhibited by heparin or genistein) [43, 45]. Inhibition of c-Src by dasatinib directs MVBs toward lysosomes for degradation, via Rab7, light chain 3-II (LC3-II), and autophagy-related gene-5 (ATG5) [46]. Dasatinib also suppresses p62 and the phosphatidylinositol 3-kinase/Akt/mTOR (PI3K/Akt/mTOR) pathway, to trigger degradative autophagy of MVBs in bladder cancer cells [46]. Dasatinib-mediated inhibition of the interaction between c-Src and ALIX can significantly decrease exosome secretion, anchorage-independent proliferation, and colony-forming activity in consensus molecular subtype 3 (CMS3) HT29 colorectal cancer (CRC) cells [30]. Src family kinases (SFK) modulate Rab27A-mediated transport of vesicles containing integrins (α3β1, α6β1) and neutrophil elastase, together with phosphorylation of cytoskeleton-associated proteins cortactin and paxillin [47]. The export of integrins and outside-in signalling allows neutrophil adhesion, crawling, and pseudopodia formation under fluid flow-induced wall shear stress [47]. Neutrophil elastase can degrade laminin in the endothelial basement membrane, to enable neutrophil transmigration from venules [47]. Non-laminar ascitic fluid shear stress increases c-Src activity, which enables adhesion adaptation and stress fibre formation in CRC cells [48]. Shear stress facilitates Src-mediated internalisation and degradation of epithelial cadherin (E-cadherin) in oesophageal cancer cells [49]. In ovarian cancer cells, shear stress promotes stem-cell-like properties (octamer-binding transcription factor 4 (OCT4), CD117, and CD44 expression), chemoresistance (ABCG2 and P-glycoprotein upregulation), and epithelial-mesenchymal transition (EMT) [50]. Dasatinib or SFK inhibitors can inhibit these c-Src-mediated processes [51].

The nSMase inhibitor GW4869 can block the exosome-induced inhibition of phosphatase and tensin homolog (PTEN), thereby increasing the therapeutic efficacy of gemcitabine in pancreatic ductal adenocarcinoma (PDAC) [52].

**Fig. 1:**
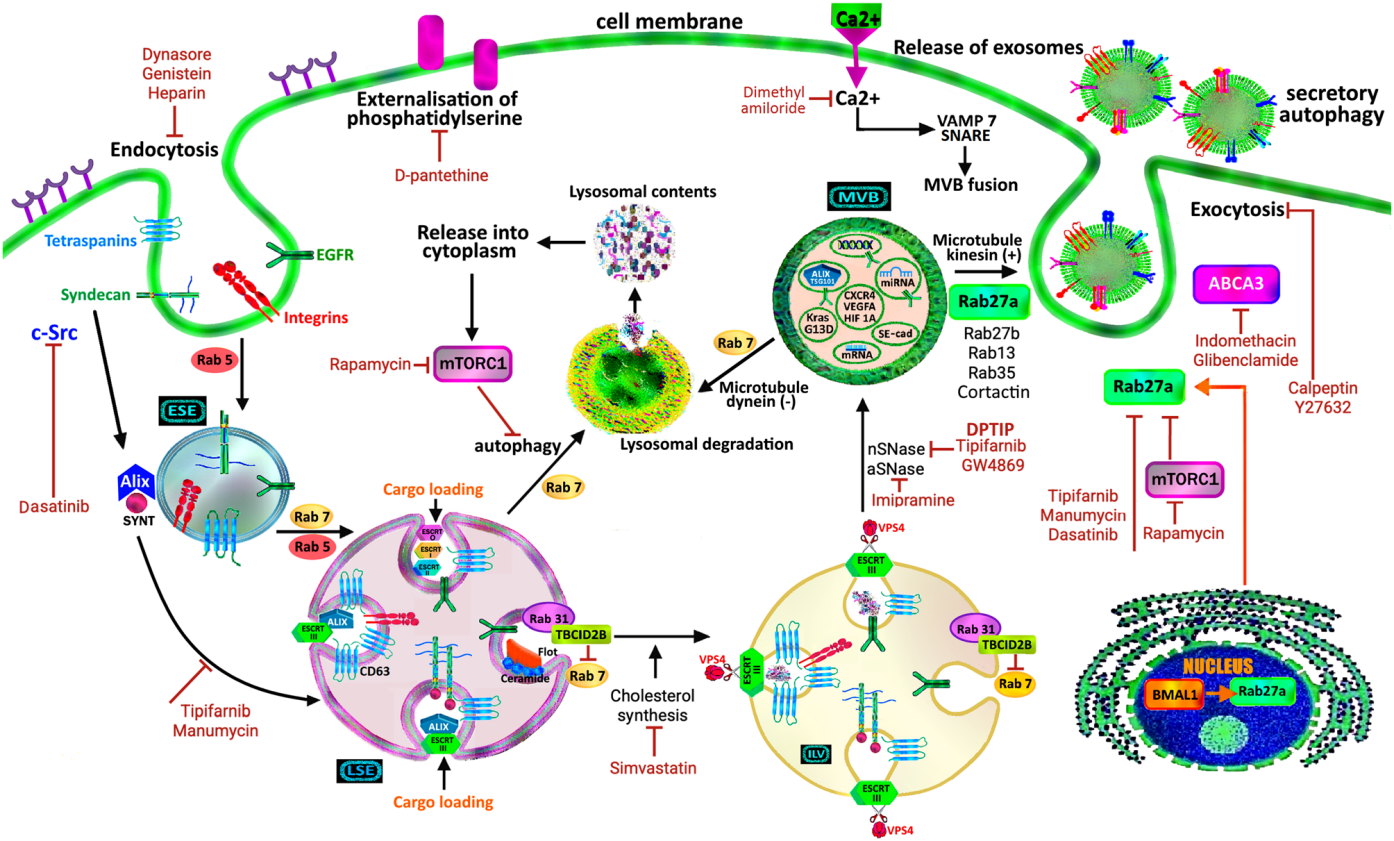
The process of exosome biogenesis. This multistep process involves endocytosis mediated by clathrin or caveolin, early sorting endosome (ESE) formation, maturation into the late sorting endosome (LSE), loading of cargo, intraluminal vesicle (ILV) development and scission by ESCRT III and VPS4, MVB formation, transport via microtubules, anchoring and fusion with the cell membrane by VAMP7 and SNARE, and exocytosis (secretory autophagy). There are four mechanisms by which ILVs are formed – the canonical ESCRT 0, I, II, III pathway; the ALIX-syndecan-syntenin-ESCRT III pathway; the Tetraspanin-ALIX-ESCRT III pathway; and the ESCRT-independent ceramide pathway, which requires activated EGFR tyrosine kinase phosphorylation of Rab31, and flotillin interactions with cholesterol and ceramide in lipid raft microdomains. The c-Src kinase phosphorylates proteins that promote exosome biogenesis and secretion, including syntenin, syndecans, and ALIX, an ESCRT-interacting molecule. Cellular Src kinase can activate ESCRT-mediated ILV formation and stimulate exosome secretion. Alternatively, late endosomes and MVBs can be directed to lysosomes by Rab7, LC3-II, and ATG5 for degradative autophagy. The recruitment of TBC1 domain family member 2B (TBC1D2B) by Rab31 inactivates Rab7, which promotes preferential MVB transport to the cell membrane and exocytosis. The release of autophagolysosome contents into the cytoplasm inhibits degradative autophagy, via a negative feedback loop with mTORC1. Exosome production can be inhibited by blockade of exosome biogenesis and trafficking at various stages (see text) [18, 21, 26–45]. Reproduced and adapted with permission from Teng et al. (2020) [20], Wei et al. (2021) [19], and Liang et al. (2024) [40]

Inhibitors of ATP-binding cassette (ABC) transporters, including non-selective NSAIDs (indomethacin) or glibenclamide, can inhibit MVB biogenesis and exocytosis at the cell plasma membrane [43]. Indomethacin increases the cytotoxicity of doxorubicin and pixantrone by the transcriptional repression of ABC subfamily A member 3 (ABCA3), thus preventing cancer cells from exporting anthracyclines via exosomes [43]. This effect does not occur with proton pump inhibitors (omeprazole), which inhibit the vacuolar-type H^+^-ATPase activity in lysosomes, or with selective cyclooxygenase-2 inhibitors (celecoxib) [43]. **(**Fig. 1).

## Environmental and cellular factors influencing exosome production

Cancer cell secretion of exosomes increases with cellular stress, such as hypoxia, hypoglycaemia, extracellular hyperglycaemia, nutritional stress, fluid shear stress, extracellular acidosis, basement membrane detachment, mitochondrial oxidative stress, nicotinamide adenine dinucleotide phosphate oxidase (NOX) activation, inflammation, loss of ATP production, generation of reactive oxygen species, the Warburg effect, and hyperthermia [50, 53–61]. Cellular stress can activate p53, which promotes transcription of the tumour suppressor-activated pathway 6 (*TSAP6*) gene that increases exosome secretion [62]. Hormones such as leptin can increase exosome secretion from tumour cells, by stimulating the expression of tumour susceptibility gene 101 (TSG101) protein in the sequential activation of ESCRT complexes [63].

## Exosomes and the hypoxic TME

The hypoxic TME induces expression of the HIF-1α subunit [64]. Hypoxia prevents hydroxylation of the α chain by HIF-prolyl hydroxylase, which has the effect of stabilising cytoplasmic HIF-1α and allowing its nuclear translocation [65, 66]. Stabilised HIF-1α levels subsequently increase, and then bind with constitutively expressed HIF-1β to form heterodimeric HIF-1 in the cell nucleus [65, 66]. The heterodimeric HIF-1 acts as a master transcription factor to activate the hypoxia response element (HRE), stimulating the transcription of over 100 validated downstream proteins [65, 66]. Activation of hypoxic signalling via dimethyloxalylglycine (DMOG), an inhibitor of HIF-prolyl hydroxylase, augments hypoxia-induced exosome secretion [67]. Hypoxia can also upregulate receptor proteins, such as epidermal growth factor receptor (EGFR) and multidrug resistance protein 1 (MDRP1), to increase exosome secretion [68].

Hypoxic TMEs can induce cancer cells to produce miR-301a-3p-rich exosomes [69]. Exosomal miR-301a-3p directly inhibits prolyl 4-hydroxylase 3 (PHD3), and thus prevents PHD3-mediated proteasomal degradation of HIF-1α [69]. The positive feedback loop between hypoxia, HIF-1α, and miR-301a-3p can enhance cancer proliferation, migration, and EMT [69], thereby enabling cancer cells to resist anoikis and survive in the hypoxic peritoneal cavity.

Exosomes can carry cargo or membrane proteins such as Src family kinases [70], MHC proteins [70], EGFR [70], integrins [70], ICAM [71], programmed death-ligand 1 (PD-L1) [42], HSPs [70], interleukin-1 (IL-1) [72], IL-6 [72], transforming growth factor-β (TGF-β) [72], KRAS [73], CXCR4 [74], tetraspanins (CD9, CD63, CD81, and CD82) [28], glycolytic enzymes [75], HIF-1α [76], soluble E-cadherin [77], and lipid metabolising enzymes [75]. Exosomes also carry nucleotides, such as mRNA of telomerase reverse transcriptase [78] or non-coding RNA (ncRNA) [79]. Exosomal cargo can affect the function and phenotype of recipient cells, such as peritoneal mesothelial cells (PMCs), fibroblasts, or adipose-derived stem cells (ADSCs), to drive every phase of PM development.

## Endocytosis of exosomes

Cells internalise exosomes by different mechanisms, depending on the type of recipient cell [80]. Platelets internalise exosomes through plasma membrane fusion, an energy-dependent process that involves destabilisation of the phospholipid bilayer [80]. Fusion occurs more readily in the acidic TME (pH < 6.5) [80], and is impaired by proton pump inhibitors, which also cause retention of acidic vesicles within tumour cells [81]. Cancer cells, fibroblasts, myeloid cells, and PMCs can internalise exosomes by micropinocytosis, which is a type of endocytosis, via membrane ruffles [80, 82]. Exosome uptake can occur via phagocytosis, such as in immune cells that engulf exosomes [80]. Endocytosis can involve membrane-associated proteins such as caveolin or clathrin, which internalise exosomes of different sizes [80]. Exosomes also carry adhesion molecules, such as activated leukocyte cell adhesion molecule (ALCAM/CD166) or CD63, to augment exosome recognition, docking or capture, and internalisation in recipient cells [83].

Upon internalising exosomes, recipient cells undergo functional and phenotypical changes triggered by the exosomal cargo. Exosomes from the primary tumour can program specific tissue of distant organs to become favourable for cancer cell implantation and metastatic progression [84]. Specific exosomal cargo, such as various integrin proteins, can assist in the formation of a pre-metastatic niche in distant organs, including the lung, liver, bone, and peritoneum [84]. Intravital imaging in an in vivo melanoma model has visualised such systemic transfer of exosomes from primary tumours to distant organs in mice [84, 85].

## CTC migration and invasion

The specific molecular mechanisms employed for tumour dissemination can be diverse [10]. The interactions between CTCs and the receptive organ microenvironment involve numerous signalling molecules, tumour-derived exosomes and cytokines, and host myeloid cells and platelets [10, 86]. CTC EMT, stemness, migration, and invasion are also augmented by mechanical forces, such as shear stress [87–89]. Shear stress can result from blood flow within the vasculature or interstitial fluid flow within the TME, as occurs in ascites with breathing, gravity, movement, or peristalsis [50, 87].

The endothelium comprises flat endothelial cells lining the inner layer of blood vessels and lymphatics, and can be continuous (either fenestrated or non-fenestrated) or discontinuous [90]. The skin, the heart, and lung capillaries have a continuous, non-fenestrated endothelium, through which molecules can move via trans-endothelial channels or caveolae-mediated transcytosis [90]. In contrast, the liver sinusoids have a fenestrated endothelium that contains clathrin-coated pits, which are crucial for receptor-mediated endocytosis, as well as a discontinuous basement membrane [90]. These features allow CTCs and leukocytes to extravasate into the space of Disse and interact with hepatocytes [90].

**Fig. 2:**
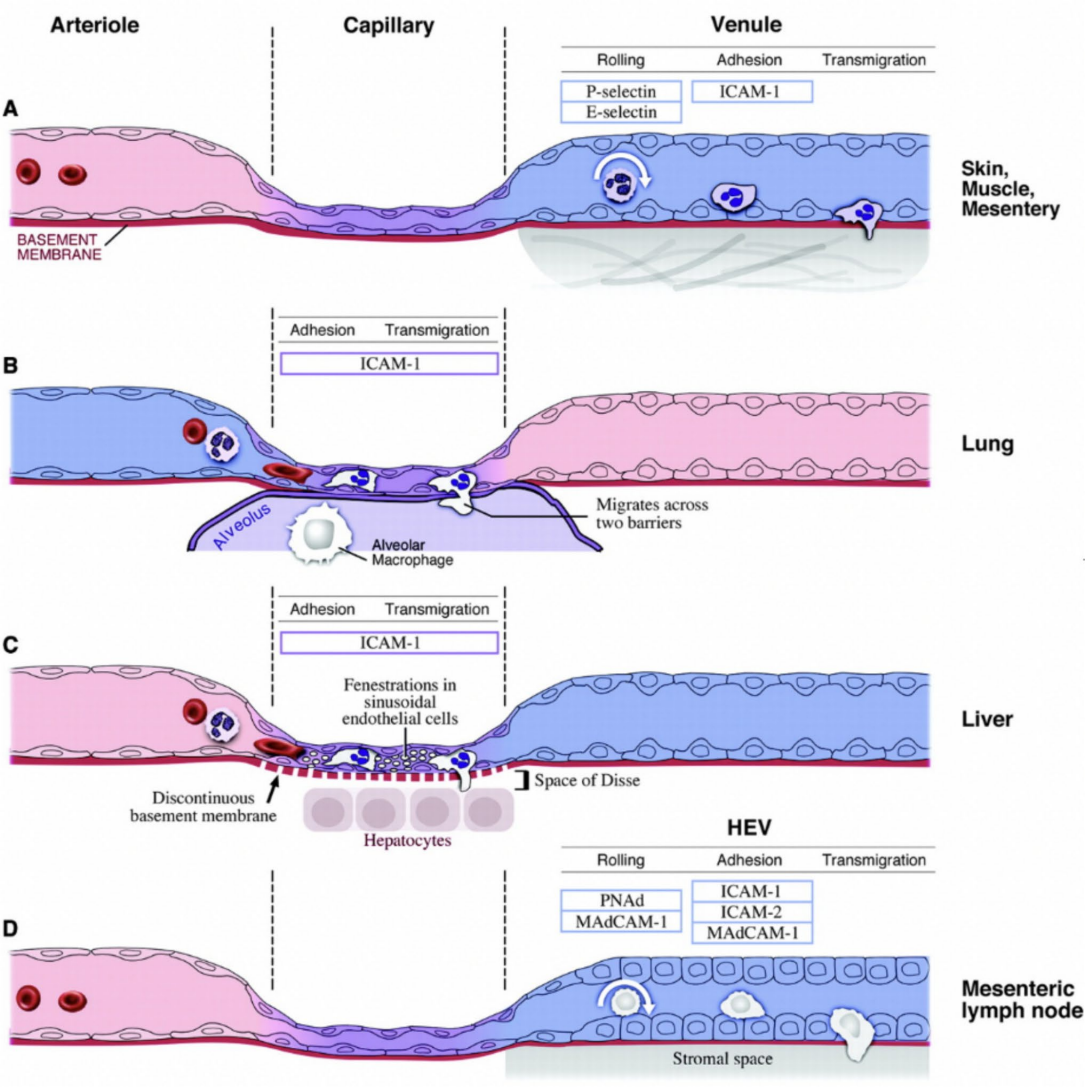
Endothelial vasculature and leukocyte extravasation. **A** In the classical multistep paradigm for leukocyte recruitment (in the skin, skeletal muscle, and mesentery), leukocytes attach to the endothelium, and then undergo rolling, firm adhesion, and transmigration through activated endothelium in venules. Rolling and adhesion involve interactions between endothelial adhesion molecules (P- and E-selectin and ICAM-1) and leukocyte counter-receptors or ligands (not shown). **B** Leukocyte extravasation in the pulmonary vasculature mostly occurs in the pulmonary capillaries. Leukocyte trafficking in the lung requires ICAM-1, but not E- or P-selectin. Leukocytes must cross two barriers (the endothelium and the epithelium) to reach the alveolar sacs. **C** Leukocyte extravasation in the liver mainly occurs in the sinusoids, which is mediated by ICAM-1. Cytoplasmic projections of the neutrophil can sense signals from hepatocytes and Ito cells, via fenestrations and gaps into the space of Disse. **D** In mesenteric lymph nodes within the peritoneum, leukocytes constitutively undergo rolling, adhesion, and transmigration across the cuboidal endothelium of HEVs. These processes involve interactions between lymphocyte L-selectin and endothelial cell peripheral lymph node addressin (PNAd) and mucosal addressin cell adhesion molecule (MAdCAM-1), and between leukocyte integrins and ICAM-1, ICAM-2, and MAdCAM-1. Reprinted from Aird WC (2007) [90]. Copyright 2007 by the American Heart Association, published by Wolters Kluwer.

The endothelial glycocalyx comprises core proteins anchored to the endothelial cell membrane, namely glycoproteins with short-branched carbohydrate side chains and proteoglycans with long, unbranched glycosaminoglycan side chains (GAG) [91]. Covering the mesh or embedded in it are soluble proteins, such as GAGs (high molecular weight hyaluronan (HMW-HA), 10^4^ kDa), proteoglycans (thrombomodulin), and extracellular superoxide dismutase and antithrombin III [91]. HMW-HA is not physically linked to core proteins but may be maintained in place by interactions with its assembly proteins, the hyaluronan synthases (HAS) or the endothelial cluster of differentiation 44 (CD44) receptor [91]. The glycocalyx is a dynamic endothelial surface layer which forms a physicochemical barrier. This glycocalyx barrier can be diminished by enzymes (heparanase-1 or hyaluronidase), cytokines (TNF-α), sepsis, hyperglycaemia, oxidised LDL, defective glycosylation, or ischaemia and reperfusion [91]. Adhesion molecules that are usually covered by the endothelial glycocalyx include selectins (E-selectin and P-selectin), integrins (integrin αVβ3*),* immunoglobulins (ICAM-1 and -2), platelet–endothelial cell adhesion molecule 1 (PECAM-1), and vascular cell adhesion molecule 1 (VCAM-1) [91]. Loss of height or degradation of the structure of heparan sulfates in the endothelial surface layer can expose the shorter adhesion molecules. The resultant docking and adhesion of circulating cells, leukocytes, and platelets lead to cell extravasation [91]. (Fig. 2).

CTCs can associate with host cells to form circulating clusters, primarily with neutrophils that are activated and attracted by the CTC-derived cytokine IL-8 (also known as chemokine (CXC motif) ligand 8; CXCL8) [92]. IL-8 also promotes neutrophil NETosis via the activation of the IL-8-CXCR2 axis, which in turn activates Src and MAPK pathways [13, 92]. The formation of CTC and neutrophil clusters is facilitated by lymphocyte function-associated molecule-1 (LFA1) [92]. Subsequently, the clusters are maintained by interactions between ICAM-1 on CTCs, Mac-1 expressed on neutrophils, integrins, and extracellular DNA from NETosis [92]. The neutrophils within clusters help the CTCs to survive vascular shear forces, and also assist in CTC vascular margination, rolling, circulatory arrest, adhesion, and extravasation [10].

**Fig. 3:**
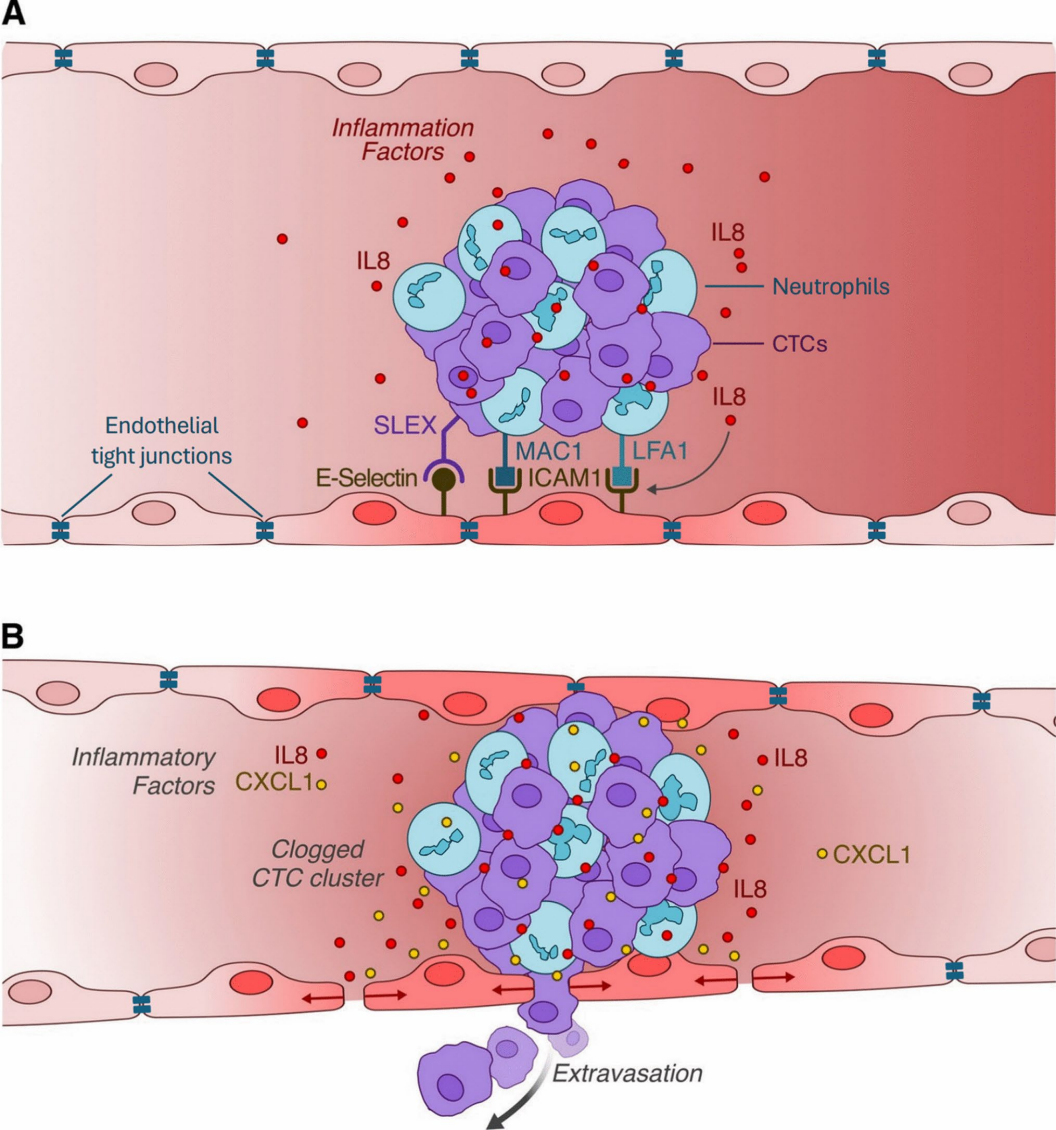
Clusters of CTCs and neutrophils. **A** Cluster adhesion to the endothelium. **B** Disruption of the endothelial barrier leads to cancer cell extravasation. Reproduced and adapted from Di Russo et al. with permission [92]

Clusters of CTCs and neutrophils are significantly associated with poorer progression-free survival in patients [92]. Adhesion is enhanced by E-selectin ligands on CTCs containing Sialyl-LewisX (SLEX) antigens binding with E-selectins on endothelial cells, as well as neutrophil LFA and Mac-1 binding with endothelial ICAM [92]. Extravasation of CTCs can involve transcellular migration through the endothelial cell or paracellular migration through fenestrations between adjacent endothelial cells [92]. Transcellular migration involves ICAM-1 and PECAM-1 adhesion molecules, podocyte formation, and reorganisation of the endothelial actin cytoskeleton [92]. Paracellular migration involves the disruption of endothelial tight junctions and adherens junctions, with the involvement of the junctional adhesion molecule family, PECAM-1, IL-8, and CXCL1 [92]. Adhesion and leukocyte transmigration in the low-velocity blood flow of the hepatic sinusoids only require ICAM, due to fenestrations in the hepatic sinusoidal endothelium and an incomplete endothelial basement membrane [90] (Fig. 3).

Similarly, omental milky spots contain incomplete peritoneal mesothelium with exposed ECM protein ligands, which can promote adhesion of exfoliated cancer cells transported through the coelom by ascites [12, 13]. Omental milky spots also contain post-capillary HEVs, which allow constitutive extravasation of neutrophils and lymphocytes mainly via paracellular diapedesis [13, 14]. Milky spots have a central hypoxic microenvironment, within which HIF-1α stimulates the formation of the pre-metastatic niche [15]. HEVs in omental milky spots express both PNAd and the MAdCAM-1, with migration of leukocytes from HEVs to milky spots requiring α4β7-MAdCAM-1 interaction but not L-selectins [16]. The structure of omental HEVs and milky spots allows rapid neutrophil recruitment and extravasation into the peritoneal cavity during septic peritonitis [13, 93]. Neutrophils that extravasate from omental milky spots undergo NETosis, to form neutrophil extracellular traps (NETs) that typically destroy microbial pathogens [93]. However, during PM, the sticky extracellular DNA traps attract, capture and shelter cancer cells from host NK cell and CD8^+^ T cell immunosurveillance [13, 93]. Omental neutrophil recruitment (7–tenfold increase) and NETosis can be remotely stimulated by cancer cell and myeloid cell-derived exosomal CXCL1, CXCL2, and IL-8 [93]. NET proteins, such as histones and myeloperoxidases, act as damage-associated molecular patterns (DAMPs) and bind to mesothelial Toll-like receptor 2 (TLR2) and TLR4 receptors [93]. This results in the mesothelial secretion of CXCL13, a chemotactic ligand for the CXCR5 receptor on IL-10-producing CD43^+^ B lymphocytes [93]. The resulting increase in IL-10 secretion promotes expansion of regulatory T cells (T_reg_) in omental milky spots [93]. Prevention of NETosis by PAD4 inhibitors (GSK484) did not affect primary ovarian cancer growth or omental neutrophil recruitment; however, GSK484 significantly inhibited CD43^+^IL-10^+^ B cell recruitment and may potentially inhibit omental metastasis in murine models of high-grade serous ovarian carcinoma (HGSOC) [93]. NETosis thus contributes to an immunocompromised premetastatic niche in omental milky spots [93].

## Formation of peritoneal metastasis

PM can arise from secondary deposits from extraperitoneal malignancies, such as breast cancer or malignant melanoma, or from transcoelomic spread from primary epithelial cancers, sarcomata or peritoneal mesotheliomas [94]. Transcoelomic spread mainly results from exfoliation of tumour cells and dissemination via ascites, as well as vascular or lymphatic extravasation of CTCs in the omentum [95]. Cancer cells may also be released into the coelom during surgical resection due to the intraoperative handling of the primary tumour, especially T_4_ cancers, or the dissection of metastatic lymph nodes and disruption of tumour-bearing lymphatics [96].

Exfoliated cells can disseminate either as single cells or as multicellular spheroids in ascitic fluid to reach distant abdominal peritoneal sites or the omentum [95]. The genesis of peritoneal tumour heterospheroids involves the recruitment of host cells, including mesothelial cells, macrophages, and CAFs, as well as interactions between EGF and EGFR, TGF-β1, integrins (α5β1), and ECM proteins [97]. Intra-peritoneal dissemination of tumour heterospheroids may require partial EMT and a stem-cell phenotype to resist anoikis [95]. Gastrointestinal malignancies, such as gastric cancer, CRC, and pancreatic cancer, may also directly invade surrounding organs [95].

Paget’s seed and soil hypothesis applies to the mechanism of PM development, as exosomes and cytokines are released by cancer cells and transported by ascites to promote metastatic organotropism, particularly in omental and peritoneal milky spots [98]. The finding that the conditioned medium from milky spots containing adipose tissue resulted in 75% greater ovarian cancer cell migration than the conditioned medium from milky spots deficient in adipose tissue confirmed the reciprocal communication between cancer cells and host cells in the peritoneal premetastatic niche [99]. Such host cells include omental ADSC, M2 macrophages, lymphocytes, activated neutrophils and platelets, and—notably—PMCs [100].

There are five potential ways in which PMC can promote transcoelomic metastasis [101].

1. PMCs express adhesion molecules on their surface, which are ligands for cell membrane receptors on cancer cells [102].
2. PMCs can acquire a senescence-associated secretory phenotype (SASP) during ageing or inflammation, and secrete exosomes, proteins, soluble factors, insoluble factors, secreted proteases, and non-protein factors to create a pre-metastatic niche and encourage transcoelomic metastasis [101].
3. PMCs undergo apoptosis under the influence of cancer cell-derived exosomes containing MMP1 mRNA, allowing cancer cell adhesion to the exposed submesothelial tissue and ECM [101, 103].
4. PMCs undergo mesothelial-mesenchymal transition (MMT) with stimulation from cancer-derived exosomes, and migrate into the basement membrane and ECM to facilitate cancer cell migration and progression [101].
5. PMCs transdifferentiate into CAFs, which enhance cancer cell survival, dispersal and progression in the coelom [101].

## The role of exosomes

**Fig. 4:**
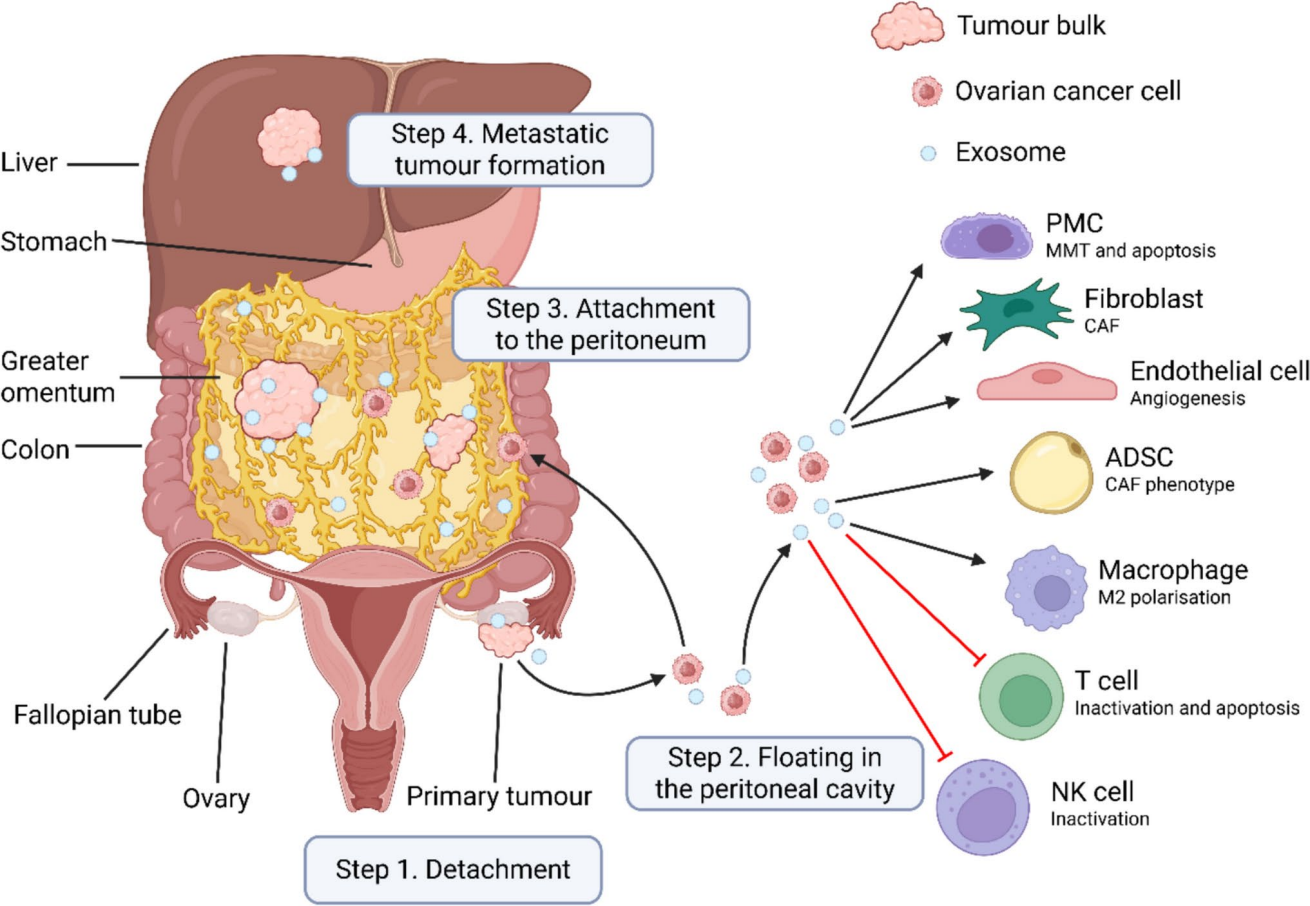
Exosomes derived from ovarian cancer promote peritoneal dissemination through various mechanisms, as illustrated in the figure. Exosomes mediate the interaction between the cancer cell and the components of the TME, to facilitate each step of ovarian cancer peritoneal dissemination. Adapted from Nakamura et al. with permission [103] and re-created in BioRender. Kim, D. (2025) https://BioRender.com/861lm6c

Cancer cells are not only the seed, but also the “ploughman” preparing the soil using cancer-derived exosomes, as hypothesised initially by Paget [1]. Exosomes play a central role in mediating communication between intraperitoneal cancer cells and PMCs [103]. Exosomes induce the peritoneum to become more receptive to transcoelomic cancer spread, by remodelling the coelomic microenvironment to form a pre-metastatic niche. Exosomes achieve this via the impairment of the mesothelial barrier, disruption of the peritoneal glycocalyx, exposure of the basement membrane and ECM, creation of the peritoneal pre-metastatic niche, MMT of PMCs, peritoneal angiogenesis, peritoneal immunosuppression, and neutrophil activation, chemotaxis and extravasation [103–105]. Exosomes encourage cancer cell ‘seeding’ in the peritoneum, by rearranging the actin cytoskeleton, formation of invadopodia, and promoting migration of both PMCs and cancer cells [37, 103]. Exosomes also prolong the anchorage-independent survival of metastatic cancer cells within the peritoneal cavity, by promoting resistance to anoikis, the reverse Warburg effect, immortalisation, mitochondrial transfer, and chemoresistance [103, 106, 107] (Fig. 4).

Cancer-derived exosomes are crucial in mediating these mechanisms of mesothelial barrier degradation. Cancer-derived exosomes can induce apoptosis of PMCs [79, 108–113]. The resultant collapse of the peritoneal mesothelium exposes the sub-mesothelial ECM, which enables cancer cell adhesion [101, 108]. Ovarian cancer-derived exosomes can increase the signalling of apoptotic and proteolytic pathways in MeT-5A PMC cells [108]. Exosomal matrix metalloproteinase (MMP) 1 mRNA and annexin A2 from ovarian cancer cells can induce apoptosis of PMCs [108, 112]. Similarly, gastric cancer cells release exosomes containing MMP2, Fas ligand, TGF-β1, miR-106a, and small nucleolar RNA host gene 12 (SNHG12), which promote the apoptosis of PMCs [79, 109–111]. Exosomal SNHG12 promotes PMC apoptosis via activation of the mitogen-activated protein kinase/extracellular signal-regulated kinase (MAPK/ERK) pathway and E2F transcription factor 7 (E2F7) upregulation [79].

## The anatomy of the peritoneal cavity and organotropism of metastases

The peritoneal mesothelium serves as a crucial anatomical barrier that can either inhibit or promote the peritoneal dissemination of cancer cells [66]. It consists of a mesothelial cell monolayer with an overlying glycocalyx, and is attached to a basal lamina and a stroma of ECM, connective tissue, capillaries, and resident fibroblasts [114]. Mesothelial cells derive from the embryonic mesoderm, and have a squamous-like appearance with microvilli extending toward the coelom [102]. They can exhibit both epithelial and mesothelial phenotypes, and express markers including various cytokeratins, E-cadherin, mesothelin, ICAM-1, calretinin, zonula occludens tight junction protein (ZO-1), β-catenin, Wilms’ tumour protein 1, and podoplanin [115]. When exposed to physicochemical damage, cytokines, extracellular acidosis, lactate, EMT transcription factors, reactive oxygen species, exosomes, or cancer cells, the epithelial-like PMC phenotype can undergo transition to a mesenchymal phenotype [115]. For example, damaged mesothelium releases CXCL2 and IL-6, which respectively recruit neutrophils to the peritoneum [116]. Neutrophil activation leads to TGF-β1 production through the induction of TNF-α [116]. Neutrophil elastase also increases TGF-β release, resulting in PMC MMT [116]. PMC MMT is characterised by decreased expression of epithelial cytokeratin and junction proteins E-cadherin and ZO-1, and enhanced expression of mesenchymal markers including N-cadherin, snail family zinc finger transcription factors (SNAIL), MMP2, α smooth muscle actin (α-SMA), vimentin, collagen-1, tenascin-C, and fibroblast-specific protein 1 (FSP-1), also called S100A4 [52, 101, 115, 117, 118].

## Mesothelial barrier and glycocalyx

The epithelial-like microvilli of PMC are covered by a thin serous layer of peritoneal fluid and glycosaminoglycans, phospholipids, proteoglycans, surfactants and coagulant precursors secreted by PMCs [102]. This slippery glycocalyx barrier protects PMCs from abrasions, infections, and cancer cell adhesion, as minimal friction enables apposing serosal surfaces to slide [102]. Microvilli significantly expand the peritoneal surface area, and possibly augment this low-friction protective barrier [102]. The glycocalyx comprises several key components, including HMW-HA and surfactant (phosphatidylcholine). HMW-HA is a linear glycosaminoglycan produced by PMCs, and represents a large hydrophilic anionic polymer with hygroscopic, rheologic and viscoelastic properties [102]. Phosphatidylcholine is also secreted by PMCs, which enhances lubrication and fluid level regulation [102]. Fluid and molecular movement across the peritoneal mesothelium can occur through the microvilli [114] via passive transport, transcellular transport, pinocytosis, plasmalemmal vesicles and stomata [102].

The PMCs on the visceral peritoneum are flat and squamous-like, with lower basal metabolic rates and tight gap junctions [102]. In contrast, the PMCs on the parietal peritoneum are cuboid, with higher basal metabolic rates [102]. Peritoneal stomata are located on the omentum, spleen, liver, falciform ligament, and the parietal peritoneum of the diaphragm and the anterior abdominal wall [102]. Peritoneal stomata around milky spots enable the movement of large molecules or cells across the mesothelial layer [102]. The ECM comprises the basement membrane and the sub-mesothelial layer, which contains various signalling molecules [102]. The ECM is exposed in omental milky spots and the cuboidal mesothelium of the parietal peritoneum on the diaphragm and the anterior abdominal wall [12, 102, 114]. In contrast, the visceral peritoneum of the intestine contains squamous-like mesothelium with tight junctions and non-exposed ECM [12, 102, 114]. The peritoneal basement membrane consists of fibronectin, laminin, and type I and IV collagen derived from PMCs [102]. The sub-mesothelial layer comprises connective tissue macromolecules derived from fibroblasts [102]. Signalling molecules sequestered in the ECM and basement membrane, including vascular endothelial growth factor (VEGF), basic fibroblast growth factor (bFGF), and latent TGF-β1, can be activated by ECM exposure, extracellular acidosis, MMPs, and matrix remodelling [102].

**Fig. 5:**
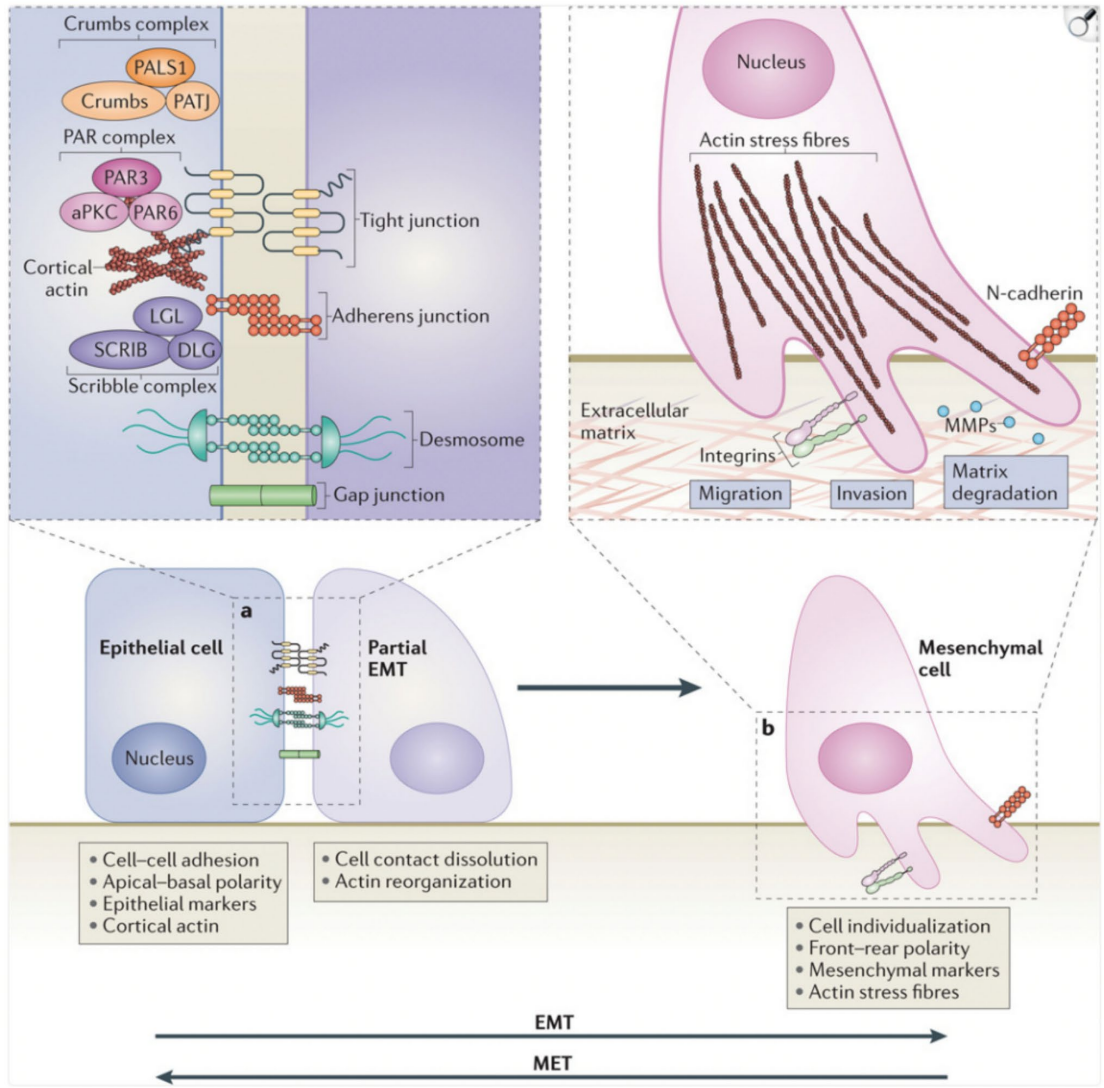
Cellular events during EMT or MMT. **a** EMT begins with the disassembly of epithelial cell-to-cell contacts, such as tight junctions, adherens junctions, desmosomes, and gap junctions. Cells lose apicobasal polarity, due to the disruption of the Crumbs, partitioning defective (PAR) and Scribble (SCRIB) polarity complexes. The expression of epithelial genes is inhibited, and mesenchymal gene expression is activated. **b** Subsequently, the rearrangement of the actin cytoskeleton promotes cell motility and invasive capability, via the formation of lamellipodia, filopodia, and invadopodia. MMPs enable the degradation of ECM proteins. The process of MET allows the cells that have undergone EMT to transition back to the epithelial phenotype. MET of disseminated cancer cells enables the formation of metastatic tumours, with phenotypes similar to those of the primary tumour. aPKC, atypical protein kinase C; DLG, discs large; LGL, lethal giant larvae; N-cadherin, neural cadherin; PALS1, protein associated with Lin-7 1; PATJ, PALS1-associated tight-junction protein. Reproduced from Lamouille et al. with permission [121]

The membrane barrier of normal epithelial cells and PMCs is maintained by intercellular junctional complexes and their respective adhesion proteins, including tight junctions (claudins and ZO-1), gap junctions (connexins), adherens junctions (E-cadherin), and desmosomes [119]. Exosomal miR-25-3p from CRC cells targets Kruppel-like factor 2 (KLF2) and KLF4 to modulate the expression of integrin ligands in endothelial cells, including ZO-1, VEGFR-2, occludin, and claudin 5 [120]**.** Loss of these junctional complexes and their ultrastructural proteins disrupts cellular apicobasal polarity, by suppressing the Crumbs, partitioning defective (PAR) and Scribble (SCRIB) polarity complexes [121]. Consequent actin cytoskeleton rearrangement and invadopodia formation enhance cellular migration and invasion, marking the progression of EMT and MMT [121]. (Fig. 5).

## Integrins

**Fig. 6:**
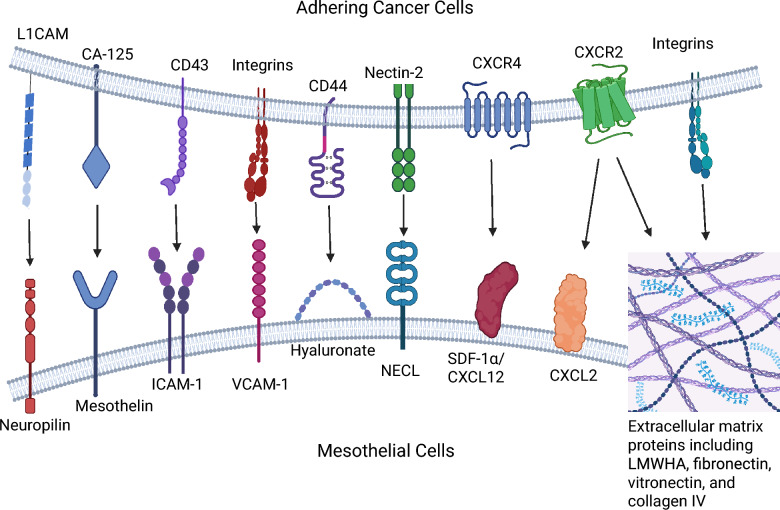
Molecular mechanisms of adhesion between exfoliated cancer cells and peritoneal mesothelium, mediating PM development. Intercellular adhesion molecule (ICAM-1). Nectin-like (NECL) family. Stromal cell-derived factor-1 (SDF-1). Adapted from Ren et al. with permission [122], and re-created in BioRender. Wilson, R. (2026) https://BioRender.com/a16hnl1

Integrins in the peritoneal mesothelium consist of two subunit combinations: α (α2, α3, α5, α6, or αv) and β (β1 or β3) [102]. Integrins are crucial for cell attachment to the ECM and intercellular interaction on the mesothelial surface (α2β1 and α3β1) [102]. PMC microvilli also express ICAM-1 and VCAM-1, which are adhesion molecules that can interact with HA, leukocytes, and cancer cells [102]. There are important interactions between HA, integrins, CD44, and hyaluronan-mediated motility receptor (RHAMM) [122]. PMCs undergoing MMT release MMPs [102], which can digest the ECM and intercellular attachments. The pathogenesis of PM involves the destruction of the mesothelial barrier by either apoptosis or MMT of PMCs, as well as the disruption of PMC HMW-HA and adherens junctions, the release of HA oligomers, and the exposure of the basement membrane and underlying ECM proteins. Intact HMW-HA (> 1000 kDa) in the mesothelial glycocalyx normally inhibits peritoneal angiogenesis and inflammation [123]. However, when its polymeric structure is disrupted, HA fragments are released. Low molecular weight HA (50–6 kDa) can promote inflammation by activating TLR-4, which in turn activates MyD88, IRAK, TRAF-6, and NF-κB, thereby enhancing the production of TGF-β1, TNF-α, and IL-6 [124]. HA fragments between 100–15 kDa stimulate cell proliferation and migration via RHAMM activation [124]. HA can bind to the cancer cell CD44, irrespective of the HA molecular size, ranging from 6 to 1800 kDa as reported in one study [124]. HMW HA-CD44 interactions promote invadopodia formation via the activation of Src-kinase, which regulates the phosphorylation of cortactin [123]. Ligand docking, disruption of the mesothelial glycocalyx and hijacking of the ensuing healing process can thus be utilised by cancer cells to encourage PM development [66]. (Fig. 6).

## E-cadherins

E-cadherin is an important type I transmembrane molecule which anchors the adherens junction between cells to intracellular catenins, the tumour suppressor adenomatous polyposis coli protein (APC), and the actin cytoskeleton [125]. E-cadherin acts as a tumour suppressor by stabilising β-catenin [125]. This prevents the nuclear translocation of β-catenin, activation of Wnt/β-catenin signalling, and induction of EMT nuclear transcription factors, including SNAIL, zinc finger E-box binding homeoboxes (ZEBs), and twist family bHLH transcription factors (TWISTs) [125]. Loss of E-cadherin can occur due to genetic or acquired causes, including loss-of-function mutations in *CDH1*, epigenetic silencing, transcriptional inhibition by SNAIL and ZEB, post-translational modification, endocytosis, or degradation [125]. Adherens junctions can be disrupted by MMPs, a disintegrin and metalloproteases (ADAMs), α-secretase, cysteine proteases (calpain), *Bacteroides fragilis* toxin, and *Helicobacter pylori* serine protease high-temperature requirement A (HtrA), which digest full-length E-cadherin and release the 80 kDa fragment as soluble E-cadherin [125].

Soluble E-cadherin promotes ECM proteolysis to encourage cancer cell invasion, and inhibits full-length E-cadherin dependent cell-cell adhesion [125]. Soluble E-cadherin can also promote the growth and survival of cancer cells by stimulating EGFR2, Wnt/β-catenin and insulin-like growth factor receptor 1 (IGF-1R) signalling and inhibiting Hippo signalling pathways [125]. Extracellular soluble E-cadherin also activates killer cell lectin-like receptor G1 (KLRG1), an immune checkpoint receptor on immune effector cells [125]. KLRG1 activation in NK and CD8^+^ T cells diminishes their cytotoxic effect on cancer cells [125]. Soluble E-cadherin exists in a positive feedback loop with EGFR [125]. Stimulation of EGFR by soluble E-cadherin increases MMPs and ADAMs, which further degrades native adherens junction E-cadherin and produces more soluble E-cadherin [125]. This positive feedback loop is directly related to the oncogenic transformation of normal epithelial cells, proliferation and invasion of cancer cells, and the extrusion and survival of cancer cells [125] in the peritoneum.

**Fig. 7:**
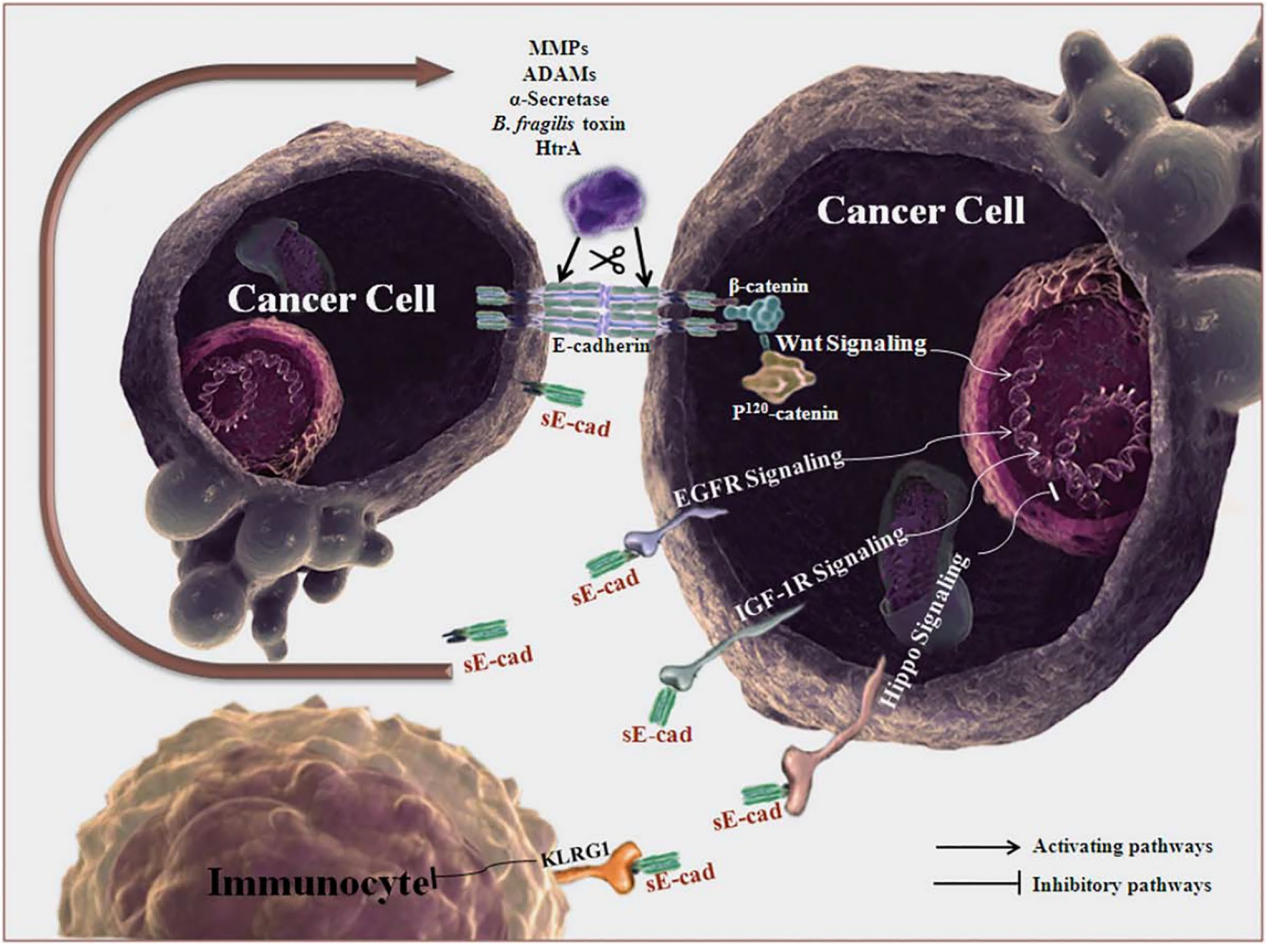
Mechanism of E-cadherin breakdown to release soluble fragments and modulate signalling pathways. Soluble E-cadherin is produced from proteolysis via MMPs, ADAMs, α-secretase, *B. fragilis* toxin and HtrA. Soluble E-cadherin activates the EGFR, Wnt/b-catenin and IGF-1R signalling pathways, and inhibits Hippo signalling. Alterations to these signalling pathways can further stimulate ADAMs and MMPs, to increase the release of soluble E-cadherin. Soluble E-cadherin also stimulates KLRG1, an inhibitory receptor on immune effector cells, to diminish the cytotoxicity against cancer cells. HtrA, High-temperature requirement protease A; MMPs, matrix metalloproteinases; ADAMs, a disintegrin and metalloproteases; sE-cad, soluble E-cadherin; EGFR, epidermal growth factor receptor; IGF-1R, insulin-like growth factor receptor 1; KLRG1, killer cell lectin-like receptor G1. Reproduced with the permission of John Wiley & Sons, from Hu et al (2016) [125]; Copyright 2016; permission conveyed through Copyright Clearance Center, Inc.

Malignant ascites is enriched with soluble E-cadherin derived from exosomes, which can promote angiogenesis by endothelial cell migration, leakage of albumin and plasma proteins, and neovascularisation [126]. Angiogenesis mediated by exosomal soluble E-cadherin has been demonstrated in breast cancer and CRC cell cultures, and is independent of VEGF [126]. Exosomes containing soluble E-cadherin are found in the ascitic fluid of patients with colon, breast, liver, endometrial, and gastric cancer [126]. (Fig. 7).

## Exosomes, mesothelial–mesenchymal transition, and CAF formation

Cancer-derived exosomes also induce MMT of PMCs [108, 109, 111, 113, 127–130]. Morphological change of PMC involving loss of polarity of PMCs from a polygonal, cobblestone shape to a mesenchymal, spindle shape (hummingbird phenotype) can occur within 48 hours after exosome exposure [108]. MMT creates new gaps between PMCs with altered morphology, which expose the basal lamina and ECM and promote cancer cell adhesion and invasion in the peritoneum [108].

Cancer-derived exosomes can induce MMT of PMCs through various mechanisms, including exosomal cargo such as MMP1 mRNA, MMP2 and TGF-β1 [108, 109, 117]. Exosomal miR-106a and miR-21-5p from gastric cancer inhibit suppressor of mothers against decapentaplegic 7 (SMAD7) and tissue inhibitor of metalloproteinases 2 (TIMP2), to activate the TGF-β pathway and elicit MMT [111, 127]. Similarly, exosomal nicotinamide N-methyltransferase (NNMT) from gastric cancer may activate the TGF-β/SMAD2 signalling pathway, inducing MMT of PMCs [128]. Conversely, low levels of exosomal miR-486-5p from gastric cancer can remove the inhibition of actin-related protein 3, SMAD2, and cyclin-dependent kinase 4 (CDK4) expression in HMrSV5 PMCs [129]. Exosome-induced MMT in gastric cancer can occur due to activation of the MAPK/ERK pathway in PMCs [109]. Exosomal transfer of lncRNA SNHG12 from cancer cells increases E2F7 expression in PMCs, which can activate MAPK/ERK signalling [79]. Exosomal annexin A2 from ovarian cancer cells promotes MMT of PMCs via the PI3K/Akt/mTOR pathway [112]. Exosomal non-coding RNA from ovarian cancer, such as the antisense transcript of spen paralogue and orthologue C-terminal domain containing 1 (SPOCD1) gene, also promote MMT of PMCs by interacting with Ras-GTPase-activating protein-binding protein 1 (G3BP1) [131].

MMT promotes the invasion of PMC into the deeper peritoneum, which further disrupts the mesothelial monolayer and allows sub-mesothelial matrix deposition [109]. Exosomal annexin A2 and TGF-β1 enhance post-MMT PMC migration [112, 117]. PMCs that have undergone MMT can also create pathways through the ECM to facilitate cancer cell migration and invasion, as discussed in the section ‘The role of exosomes’ (Table 1). Such mesothelial barrier injury enables the transcoelomic spread of metastatic cancer cells by successful implantation and invasion of the peritoneum [109].

**Table 1:** A summary of key exosomal cargoes involved in PM development

Exosomal cargo	Effect	Donor cell	Reference
Src family kinases	Promote exosome assembly and secretion, invadopodia formation		[30, 31, 123]
Integrins	Promote pre-metastatic niche, EMT in tumour heterospheres, adherence of cancer cells to PMC ECM, TGF-β release, PD-L1 expression, intercellular transfer of MDRP1/P-glycoprotein		[22, 70, 97]
EGFR	Promote assembly of tumour heterospheres and pre-metastatic niche formation		[70, 97]
ICAM	Maintain CTC-neutrophil clusters		[71, 92]
IL-6	Recruit neutrophils to the peritoneum		[72, 116]
IL-8	Enhance CTC-neutrophil clusters and promote NETosis		[92]
TGF-β	Promote pre-metastatic niche formation, TME immunosuppression, and MMT of PMCs		[22, 72, 109, 152]
Glycolytic enzymes	Facilitate anaerobic glycolysis and lactate formation (p-PDH‐E1α, LDHA), and promote resistance to anoikis		[75, 160]
HIF-1α	prepare the omental pre-metastatic niche, promote omental PMC MMT, milky spot T cell exhaustion and M2 Mø expression of PD-L1, Arg1, IL-1β, IL-6, and CCL5	Gastric Cancer	[160]
Stabilizing HIF-1α by ncRNA	Transfer of exosomal miR-301a-3p inhibits PHD3 and stabilizes HIF-1α, promoting GC proliferation, invasion, pseudopodia formation, migration, EMT and PM	Gastric Cancer	[69]
PD-L1	Enable immunological privilege in PMC milky spots for CSC survival	Gastric Cancer	[134, 160]
Soluble E-cadherin	Promote cancer cell growth and survival via EGFR, Wnt/b-catenin, IGF-1R, and Hippo signalling		[125, 126]
Lipid metabolising enzymes	Reprogram cancer cell metabolism in peritoneal omentum, and enable reverse Warburg effect via cancer-associated adipocytes (CAA)		[52, 75]
ALCAM/CD166	Promote cancer cell adhesion to peritoneal mesothelium		[83]
MMP	disrupt peritoneal mesothelium via PMC apoptosis, enable disaggregation of tumour heterospheres, promote ligand binding of mesothelial ECM proteins to cancer cell integrins, peritoneal pre-metastatic niche		[108, 109]
CD44	Promote PMC MMT, PMC release of MMP9, and cleared areas of mesothelium	Ovarian cancer	[103]
Annexin A2	Disrupt peritoneal mesothelium via PMC apoptosis, PMC MMT and degradation of ECM via PI3K/AKT/mTOR pathway	Ovarian cancer	[112]
SNHG12	Disrupt peritoneal mesothelium via PMC apoptosis, and promote MMT of PMCs	Gastric cancer	[79]
Fas ligand	Disrupt peritoneal mesothelium via PMC apoptosis	Gastric cancer	[109]
miR-106a	Disrupt peritoneal mesothelium via PMC apoptosis	Gastric cancer	[110, 111]
miR-301a-3p	Promote cancer cell proliferation, migration, and EMT	Gastric cancer	[69]
miR-25-3p	Rearrange actin cytoskeleton and promote invadopodia formation		[120]
miR-21-5p	MMT of PMCs by activating TGF-β/SMAD pathways, via inhibition of SMAD7	Gastric cancer	[127]
NNMT	Promote MMT of PMCs via TGF-β/SMAD2	Gastric cancer	[128]
miR-181a-5p	Promote formation of pro-angiogenic CAFs	CRC	[52]
miR-146a-5p	Promote formation of CAFs	CRC	[52]
miR-155-5p	Promote formation of CAFs	CRC	[52]
HSPC111	Promote lipid metabolism in CAFs via ACLY/acetyl-CoA/CXCL5 axis	CRC	[52]

**Fig. 8:**
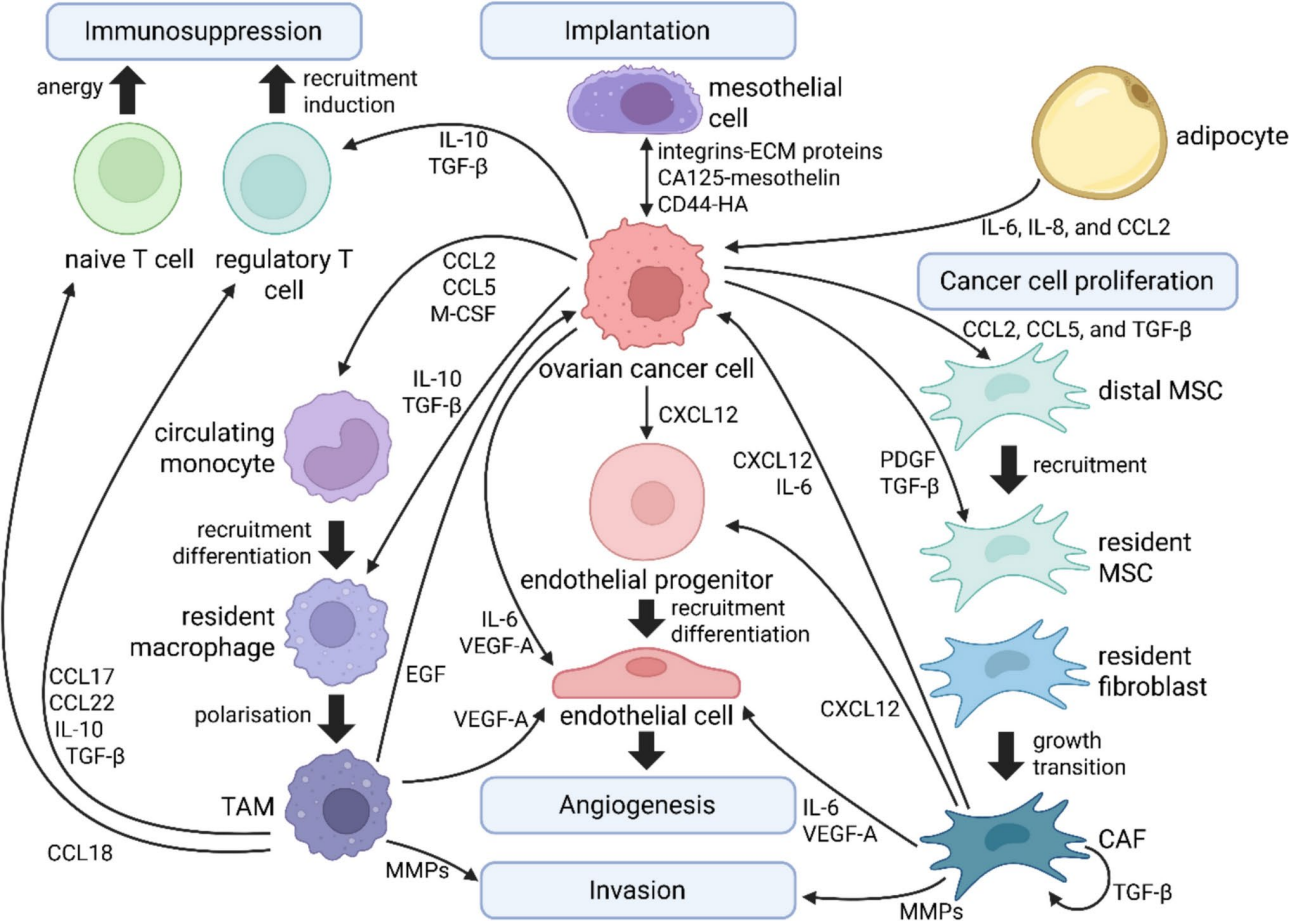
The development of PM from ovarian cancer relies on the interplay between cancer cells, PMCs and CAFs, endothelial cells, and immune cells. Adapted from Naora et al. with permission [132], and re-created in BioRender. Kim, D. (2025) https://BioRender.com/j1gdxqi

Creation of CAFs results from exosomal transfer of cytokines and miRNA from cancer cells to PMCs and stromal cells, including resident fibroblasts, adipocytes, and myeloid-derived suppressor cells [52, 132]. (Fig. 8) CAFs display almost all the properties of normal fibroblasts, but have higher proliferative rates and secrete more cytokines, matrix proteins, and immunomodulatory factors to affect the TME [52]. CAFs are heterogeneous across tumour development stages, and exhibit distinct phenotypes in different parts of tumour tissue [52]. CAFs are classified into four categories: Reactive CAFs, myofibroblast CAFs, inflammatory CAFs, and antigen-presenting CAFs [52]. Myofibroblast CAFs enhance ECM remodelling by producing collagen and modulating mechanical conduction [52]. Inflammatory CAFs modulate the immune response, whilst antigen-presenting CAFs activate CD4^+^ T cells for specific antigens [52]. These three CAF types, when combined, can substantially augment the proliferation, migration, invasion, metastasis, and chemoresistance of cancer cells [52]. Exosomal miR-181a-5p, miR-146a-5p, and miR-155-5p from CRC can expedite the transdifferentiation of normal fibroblasts into CAFs [52]. These exosomal miRNAs also increase the secretion of cytokines from CAFs, including IL-6, TNF-α, TGF-β, and CXCL12 [52]. Exosomal HBV pre-S2 trans-regulated protein 3 (HSPC111) from CRC can modify lipid metabolism in CAFs by phosphorylating ATP citrate lyase (ACLY), thereby increasing acetyl-coenzyme A synthesis and CXCL5 expression and secretion [52].

CAFs can also arise from mesenchymal stem cells (MSCs), such as in gastric cancer [133]. The transdifferentiation of MSCs into CAFs requires the hedgehog signalling pathway, which can be inhibited by monoclonal antibody (5E1) targeting hedgehog ligands [134]. Once formed, MSC-derived CAFs can be recruited from the bone marrow to the dysplastic gastric tissue, in a TGF-β- and CXCL12-dependent manner [133]. MSC-derived CAFs stimulate cancer progression via IL-6 and DNA hypomethylation [133]. (Fig. 8).

## Mesenchymal-type tumours, peritoneal metastasis and exosomes

A predilection for PM development characterises a subset of cancers, such as diffuse gastric cancer (DGC) [135], CMS4 CRC [136], signet ring cell cancers [137], HGSOC [97], and PDAC [138]. The risk of PM development from gastric cancer increases with mutations in *TP53*, *CDH1*, *TAF1*, and *KMT2C* genes, most of which are exclusive to DGC [135]. DGC exhibits poorly differentiated histology [135] and lacks E-cadherin, which normally maintains cellular polarity and intercellular attachments through adherens junctions [139]. The loss of E-cadherin may be due to germ cell *CDH1* mutations in hereditary DGC, or *H. pylori*-derived serine protease HtrA and CagA causing somatic cell disruption of E-cadherin in sporadic DGC [139, 140]. The resultant development of mesenchymal-like cancer cells in DGC [139] promotes invasion into the muscularis propria and transcoelomic spread. The mesenchymal subtype of gastric cancer exhibits greater expression of immune checkpoint T-Cell Immunoglobulin and Mucin Domain-Containing Protein 3 (TIM-3), its ligand galectin-9, V-domain Ig suppressor of T cell activation (VISTA), and TGF-β [135]. Exosomal TIM-3, galectin-9, VISTA, and TGF-β represent potential targets for immunotherapy [135, 141]. DGC is associated with significantly lower levels of host cytotoxic lymphocytes, NK cells, myeloid dendritic cells, and resident peritoneal fibroblasts, potentially due to the reprogramming of the TME [135].

Gastric cancer also exhibits different molecular subtypes with implications for targeted therapy options [142]. The Cancer Genome Atlas (TCGA) classification of gastric adenocarcinoma identifies four molecular subtypes [142]. Epstein-Barr virus-positive (EBV^+^) tumours have recurrent *PIK3CA* mutations, extreme DNA hypermethylation, and amplification of *JAK2*, *PD-L1*, and *PD-L2* [142]. Microsatellite unstable (MSI) gastric cancers have high mutation rates of genes encoding targetable oncogenic proteins [142], and demonstrate the best 5-year overall survival rates [143]. Genomically stable tumours are typically found in DGC, and display mutations of *CDH1* and *RHOA* [142]. Tumours with chromosomal instability demonstrate significant aneuploidy and focal amplification of tyrosine kinase receptors [142]. Similarly, the Asian Cancer Research Group (ACRG) classification of gastric cancer characterises four distinct molecular subtypes, based on principal component analysis on the gene expression data set [143]. These are the MSI^high^, the microsatellite stable/tumour protein 53-active (MSS/TP53^+^) and TP53-inactive tumours (MSS/TP53^−^), and the mesenchymal-type gastric tumours that are characterised by EMT (MSS/EMT) [143]. MSS/EMT gastric cancers portend the worst prognosis, and have a greater risk of peritoneal seeding (64%, *n* = 41/64) than all other subtypes (23%, *n* = 39/172) [143].

Similarly, CRC is divided into four molecular subtypes: CMS1–4 [144]. CMS4 CRC exhibits a mesenchymal phenotype, characterised by prominent TGF-β signalling, integrin interaction with matrix proteins, angiogenesis, and the recruitment of innate immune cells, which creates an inflammatory TME [144, 145]. Mesenchymal markers of the CMS4 subtype include platelet-derived growth factor receptor-α (PDGFR-α), PDGFR-β, PDGF-C, and KIT receptor tyrosine kinase [144]. In one study, CMS4 CRC was identified in 82.7% (43 of 52 cases) of PM from CRC [136]. This is an over-representation of the CMS4 subtype of CRC, which typically accounts for only 25% of overall primary CRCs [136]. In contrast, the proportion of CMS4 CRC causing liver metastasis does not exceed 35% [136, 146, 147]. The mesenchymal CMS4 CRC subtype exhibited lower oxygen consumption and reduced mitochondrial content [136]. Metabolomic analysis shows increased lactate and tricarboxylic acid cycle intermediates in CMS4 CRC cells, suggesting a reduced capacity for oxidative phosphorylation [136]. CMS4 CRC cells also display increased rates of *MYOF* exon 17 inclusion, which may alter the diversity of exosome proteomes and promote CRC metastasis [148].

EOC can be further divided into five histological subtypes: HGSOC, low-grade serous ovarian cancer (LGSOC), clear-cell ovarian cancer, endometrioid ovarian cancer, and mucinous ovarian cancer [149]. HGSOC comprises nearly 80% of all EOCs [149]. In the Australian Ovarian Cancer Study and then in the TCGA Research Network study, HGSOC was categorised as “immunoreactive”, “differentiated”, “proliferative”, and “mesenchymal” subtypes based on gene expression analysis [150]. Mesenchymal-type HGSOC is associated with cisplatin resistance and poor overall survival; however, when combined with proliferative HGSOC, patients with PM involving these tumours may benefit from treatment with the VEGF inhibitor bevacizumab [151]. The resistance to platinum-based chemotherapy in mesenchymal HGSOCs with an EMT phenotype may be attributed to the absence of *BRCA1/2* mutations or the induction of drug efflux transporters [151].

**Fig. 9:**
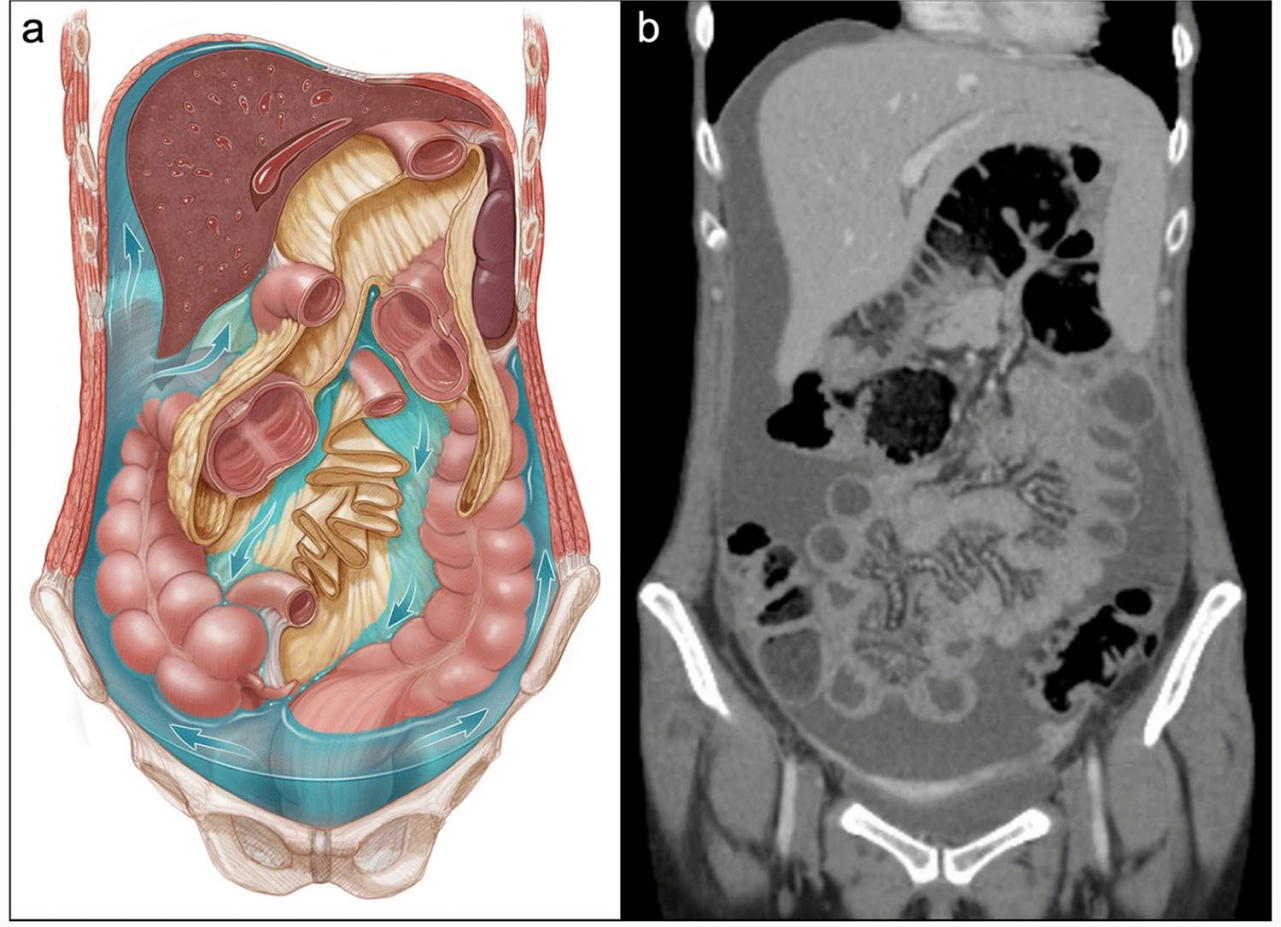
Flow dynamics of ascitic fluid in the peritoneum. **A** In the abdominal cavity, ascitic fluid initially collects in the pelvic recesses due to gravity. Accumulated fluids then flow upwards, pushed by the pressure gradient toward the Morison pouch and the right subphrenic space, along the right paracolic gutter. Ascitic fluid also flows upwards along the left paracolic gutter, albeit the phrenicocolic ligament limits this. **B** Coronal CT image depicting PM secondary to metastatic gastric cancer, with ascites in the pelvis, right paracolic gutter and right subphrenic space. Reproduced from Gonzalez et al. with permission [155]

Unlike many epithelial cancers that develop haematogenous metastases, EOC mainly disseminates in the peritoneum via transcoelomic metastasis [152]. Metastatic EOC cells in the peritoneal cavity survive as single cells or multicellular spheroids in ascitic fluid [152]. Cancer cells can undergo anoikis from insufficient cell–matrix interaction in ascites [152]. However, EMT induced by TGF-β in ascitic fluid can enable mesenchymal-type HGSOC to resist anoikis [152, 153]. Cancer cells in ascites can disseminate in the abdominal cavity due to the circulation of ascitic fluid by gravity (Krukenberg tumours), peristalsis, and diaphragmatic excursions [154]. Gravity and sub-diaphragmatic pressure together determine the flow of ascitic fluid within the peritoneal cavity, which directly affects the location of PM in ovarian cancer [154, 155]. (Fig. 9).

Cancer cells can attach to omental milky spots on the omentum within minutes to hours of their release into the coelom [156]. This process involves numerous chemotaxis and adhesion mechanisms, including:

1. Increased mesothelial expression of ICAM in milky spots compared to other mesothelial sites on the omentum, a receptor for CD43 and MUC1 on cancer cells [90, 122].
2. EMT-mediated upregulation of α5β1 integrin (a receptor for fibronectin) enabling cancer spheroid attachment to the peritoneal mesothelium [152].
3. HGSOC expression of cancer antigen 125 (CA125), a glycoprotein that binds the mesothelin of PMCs [157].
4. EOC CXCL12-C-C motif chemokine receptor 4 (CCR4) [157], and by milky spot macrophage expression of CCR1 ligand [158], which promote EOC cell homing, adhesion, invasion and proliferation at omental milky spots.

**Fig. 10:**
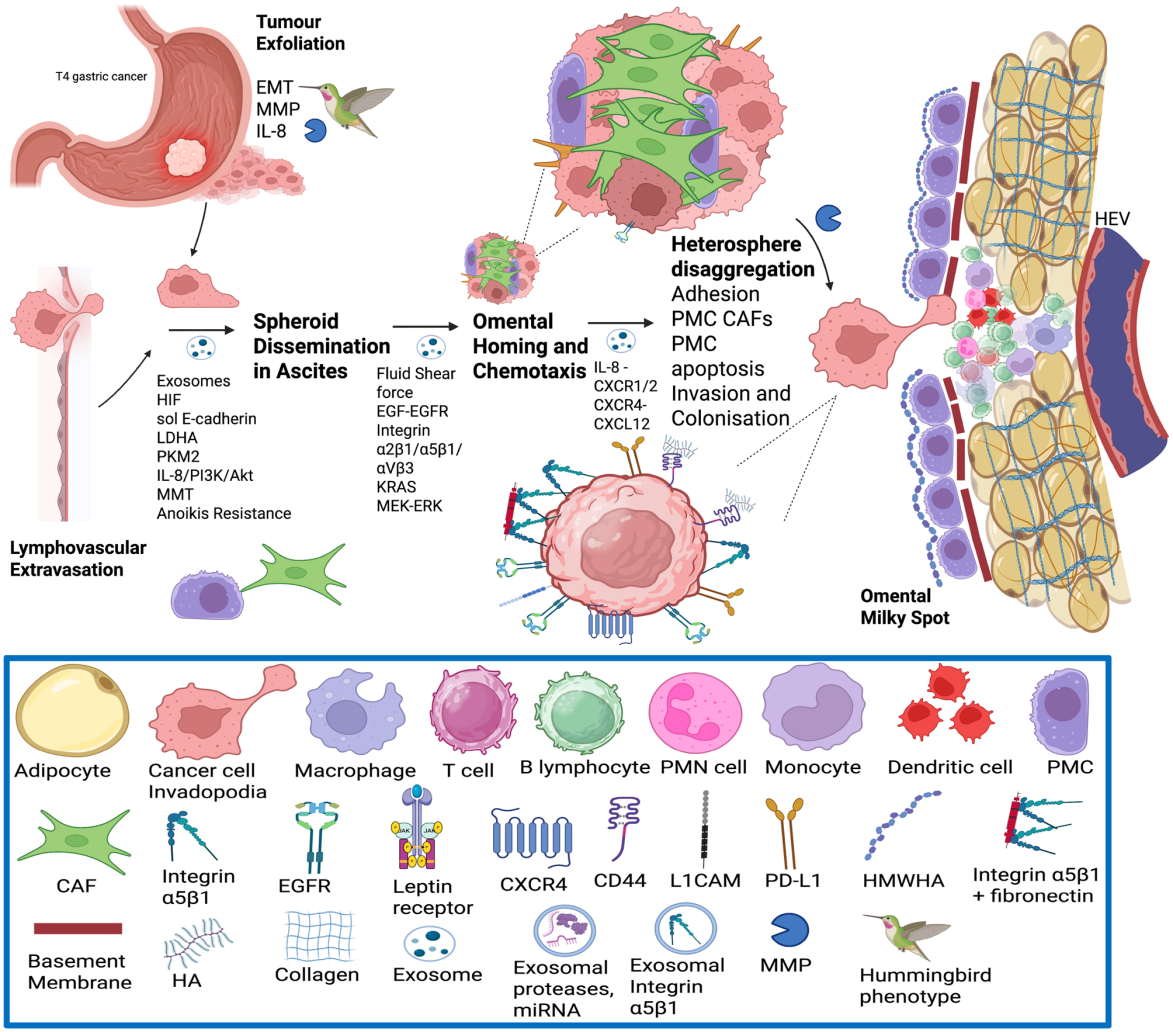
Mechanism of peritoneal dissemination from gastric cancer to omental milky spots. Anoikis resistance mediated by EMT, metalloproteases, integrins, exosomes, HIF, EGFR activation, glycolytic enzymes, and metabolic coupling with CAFs from transformed PMCs and resident fibroblasts [52–167]. Created in BioRender. Wilson, R. (2025) https://biorender.com/z7wh46x; https://BioRender.com/03533mz

**Fig. 11:**
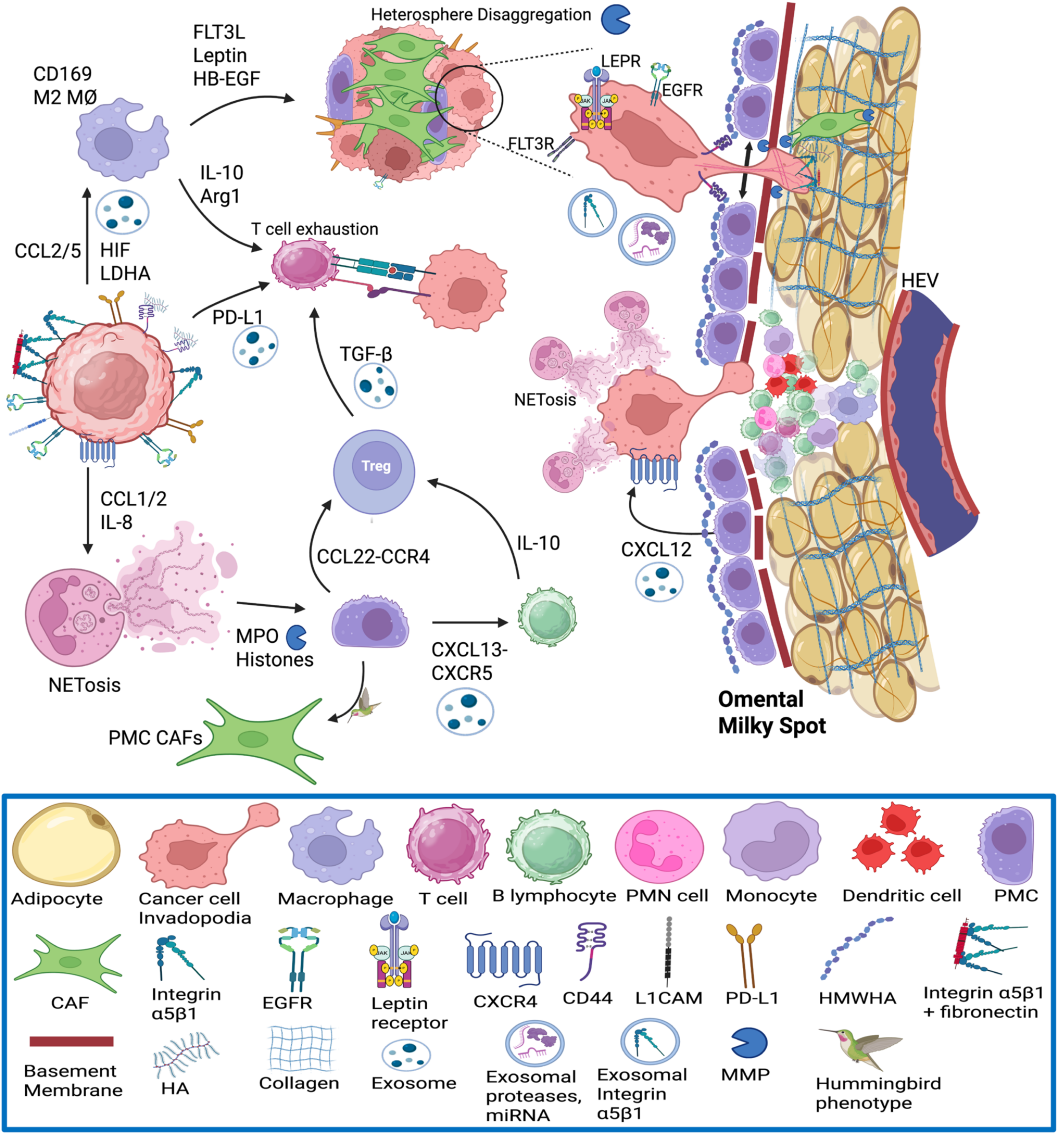
Metastatic Organotropism to Omental Milky Spots. The migration of gastric cancer, CRC, and EOC cells to the omentum involves apoptosis of PMC, adhesion of tumour cells to the underlying basement membrane and formation of invadopodia. Release of exosomes containing integrin α5β1, miRNA and proteases leads to clearance of PMCs and access to underlying ECM proteins including collagen, fibronectin and HA. Omental milky spots contain HEVs which facilitate extravasation of neutrophils, lymphocytes, and macrophages. Remote recruitment and activation of neutrophils by IL-8 promotes NETosis. This protects cancer cells from T cell immunosurveillance, and contributes to T cell exhaustion. PMCs secrete CXCL12 which enhances cancer cell homing to omental milky spots via CXCR4, and CCL22 which promotes Treg expansion. Macrophages are polarised to the M2 subtype by cancer cell-derived CCL2/5 and exosomal LDHA and HIF. M2 macrophages secrete leptin, HB-EGF and FLT3L which activates their cognate receptors on cancer cells in ascitic heterospheres, leading to activation of JAK2/STAT3 signaling and release of MMPs. Subsequent disaggregation of heterospheres and formation of invadopodia enables cancer cell adhesion to the omental mesothelium, proteolysis and invasion of the underlying stromal ECM. Release of TGF-β, PD-L1, IL-10 and Arginase-1 (Arg1) by M2 macrophages and Treg cells leads to failure of T-cell killing of cancer cells, and formation of the immune-privileged niche in omental milky spots [52–167]. Created in BioRender. Wilson, R. (2025) https://BioRender.com/hyepec2; https://BioRender.com/03533mz

Upon contact with a receptive peritoneal niche, MMP2 and MMP14 on the surface of EOC cells can degrade fibronectin, vitronectin, and collagen IV in the mesothelial basement membrane, thereby enhancing further integrin docking, adhesion, and invasion [157]. The release of MMP2 from cancer cells and MMP9 from host mesothelial cells enables the disaggregation of tumour heterospheres and facilitates invasion at the metastatic site in the omentum. Transfer of exosomal CD44 from EOC stimulates PMC MMT, release of MMP9 from PMC, and clearance of the peritoneal mesothelial barrier [103]. Tumour MMP14 also digests fibrillar collagens of the sub-mesothelial stromal matrix, which promotes invasion into deeper layers of the peritoneum and angiogenesis [157]. Once cancer cells establish a lipid fuel source from adipocytes for mitochondrial β-oxidation and a vascularised stroma for oxygen supply, they can differentiate via mesenchymal-to-epithelial transition (MET) and proliferate in the adjacent omentum, with phenotypes similar to those of the primary tumour [121, 152]. (Figs. 10, 11).

However, in the hypoxic centre of milky spots, differentiated cancer cells—but not cancer stem cells—are removed by cytotoxic macrophages [15]. Hypoxia is a feature of peritoneal milky spots, to which detached cancer cells are attracted and adhere. HIF-1α in peritoneal milky spots helps to maintain metastatic DGC cells in an undifferentiated state [15]. In vitro evidence shows hypoxic gastric cancer cells express stem cell-related proteins (OCT4 and nestin) and EMT markers (leucine-rich repeat-containing G protein-coupled receptor 5 (LGR5) and CD44), but have minimal co-expression of epithelial markers (mucin 5ac and mucin 6) [15]. This represented an *immunologically privileged* stem cell niche in peritoneal milky spots analogous to that of the hypoxic gastric glands [134]. Hypoxia can intensify the malignant transformation of gastric epithelial cells triggered by chronic *H. pylori* infection [159]. The hypoxic niche of peritoneal milky spots enhanced self-renewal ability in DGC cells and the generation of tumour spheres with EMT properties, which promotes further peritoneal metastatic dissemination [15].

Exosomes derived from lactosylceramide alpha‐2,3‐sialyltransferase positive (ST3G5^+^) DGC cells are enriched in HIF-1α and lactate dehydrogenase A (LDHA), which are internalised by milky spot macrophages and dendritic cells expressing sialic acid‐binding Ig‐like lectin 1 (CD169/SIGLEC1) [160]. The endocytosis of these DGC-derived exosomes by host macrophages stimulated their release of CC‐chemokine ligand 5 (CCL5), activation of STAT3 signalling, anaerobic glycolysis and lactate formation, phosphorylated pyruvate dehydrogenase‐E1α, restricting the synthesis of acetyl‐coenzyme A in milky spot macrophages, MMT in PMCs and their transformation into α-SMA^+^ CAFs [160]. Glycolysis and lactate formation by M2 macrophages lead to the release of immune checkpoint molecules (PD-L1), inflammatory cytokines (IL-1α, IL-6), NF-κB, arginase 1 (Arg1), and T cell exhaustion and apoptosis in milky spots, furthering the ability of DGC cells to escape host immune editing [160]. Blockade of the interaction between CCL5 and its receptor CCR5, achieved through treatment with maraviroc, prevented ST3G5^high^-cExo-mediated PMC MMT, T-cell suppression and DGC metastasis in omental milky spots in vivo [160]*.* Expression of ST3G5 in resected primary gastric cancer specimens was associated with an increased risk of postoperative peritoneal recurrence in patients [160]. Furthermore, tumour-associated macrophages secrete CCL5 that mediates the NF-κB-p65/STAT3/CSN5/PD-L1 pathway [161]. PD-L1 expression subsequently increases in the TME of both HT29 (MSS) and HCT116 (MSI) CRC cells in vitro, irrespective of microsatellite status [161]. This is important in the treatment of patients with MSS mesenchymal-type cancers, such as DGC and CMS4 CRC, which typically have poor responses to immune checkpoint inhibitors compared to those with MSI^high^ gastric cancer or CMS1 CRC [161–164]. (Table 1).

When compared to primary HGSOC and solid metastatic HGSOC cells, ascitic tumour cells express greater paxillin and establish focal adhesions [97]. In both HGSOC and LGSOC, ascitic tumour cells display greater capability for adhesion, invasion, and mesothelial clearance [97]. Compared to matched cells from primary tumours and solid metastases, HGSOC ascitic tumour cells exhibit upregulated integrin α5 (ITGA5) and β3 levels [97]. Both tumour ITGA5 mRNA and protein levels are associated with a significantly poorer 5-year overall survival in patients with serous ovarian cancer [97]. The majority of ovarian cancer cells adhering to the omentum and mesentery in PM are cells containing ITGA5 [97]. Knockdown of ITGA5 in SKOV3 ovarian cancer cells significantly reduces the capability for adhesion or forming ascitic spheroids [97]. CAFs recruit ITGA5-enriched ascitic tumour cells to produce aggregates of heterotypic ascitic spheroids, which are invasive and chemoresistant cancer cell populations crucial in anoikis resistance and metastatic dissemination [97, 165]. CAFs in the core of ascitic spheroids contain α-SMA, PDGFR-β, or prolyl 4-hydroxylase [97]. Ascitic spheroids help maintain ascitic tumour cell ITGA5 expression by CAF-derived EGF [97]. ITGA5 expression in ascitic heterospheroids from patients with HGSOC could be significantly reduced by EGF-neutralising antibodies in the TME [97]. The pro-metastatic effects of α5β3 integrin may be propagated by exosomes, which can transfer α5β3 integrins from cancer cells to other stromal or cancer cells [166]. Upon the uptake of exosomes containing α5β3 integrins, the de novo expression of α5β3 integrin in recipient cells enhances their adhesion and migration capabilities [166] (Figs. 10, 11).

Ovarian cancer heterospheroid formation is augmented by the upregulation of the KRAS, MEK-ERK, and EGFR signalling pathways [122]. HGSOC exhibit a relative abundance of ascitic CAFs and the associated ascitic spheroids [97], which is associated with the rapid progression of PM and worse prognosis in HGSOC [167] compared to LGSOC. Both HGSOC and LGSOC can exhibit either complete or incomplete EMT phenotypes [168]. Primary HGSOC has four molecular subtypes: Immunoreactive, mesenchymal, proliferative, and differentiated [144]. However, regardless of the phenotype of the primary ovarian tumour, PM arising from ovarian cancer exhibit mesenchymal properties [144]. Primary peritoneal cancer also typically displays the mesenchymal phenotype, which suggests mesenchymal transformation represents a key trait of peritoneal malignancy [144].

## Conclusion

The pathogenesis of PM involves exosomes, which enhance the formation of the pre-metastatic niche in the peritoneal microenvironment, particularly in milky spots. The exosome-mediated disruption of the peritoneal mesothelial barrier and MMT enables transcoelomic cancer dissemination, as well as cancer cell homing, adhesion, invasion, angiogenesis, stromal recruitment, ascites formation and suppression of host immunosurveillance. Mesenchymal molecular subtypes of epithelial cancers, including CMS4 CRC, HGSOC and DGC (MSS/EMT), demonstrate a predilection for PM. Paget’s seed and soil hypothesis applies to the mechanism of PM development, as exosomes and cytokines are released by cancer cells and transported by ascites to promote metastatic organotropism, particularly in omental and peritoneal milky spots. This is enhanced by the recruitment and extravasation of host neutrophils and macrophages from milky spot HEVs, and subsequent NETosis and M2 macrophage polarisation. Understanding the molecular machinery underlying exosome biogenesis, signal transduction, and cellular response offers potential for future targeted treatments and preventive therapies in PM.

## Data Availability

No datasets were generated or analysed during the current study.

## Abbreviations

ALCAM/CD166: Activated leukocyte cell adhesion molecule
APC: Adenomatous polyposis coli
ADAMs: A disintegrin and metalloproteases
ADSC: Adipose-derived stem cells
ALIX: Apoptosis-linked gene-2-interacting protein X
ACRG: Asian Cancer Research Group
α-SMA: α Smooth muscle actin
ABC: ATP-binding cassette
ABCA3: ATP-binding cassette subfamily A member 3
ACLY: ATP citrate lyase
aPKC: Atypical protein kinase C
ATG5: Autophagy-related gene-5
bFGF: Basic fibroblast growth factor
CAFs: Cancer-associated fibroblasts
CCL5: CC‐chemokine ligand 5
CCR4: C-C motif chemokine receptor 4
CTCs: Circulating tumour cells
CD44: Cluster of differentiation 44
CMS4: Consensus molecular subtype 4
DGC: Diffuse gastric cancer
DLG: Discs large
DPTIP: 2,6-dimethoxy-4-(5-phenyl-4-thiophen-2-yl-1H-imidazole-2-yl)-phenol
E2F7: E2F transcription factor 7
ESCRT: Endosomal sorting complex required for transport
EGF: Epidermal growth factor
E cadherin: Epithelial cadherin
EMT: Epithelial–mesenchymal transition
EOC: Epithelial ovarian carcinoma
EBV: Epstein–Barr virus
ECM: Extracellular matrix
FSP-1; S100A4: Fibroblast-specific protein 1
GAG: Glycosaminoglycan
HSPC111: HBV pre-S2 trans-regulated protein 3
HSP: Heat shock protein
HMW-HA: High molecular weight hyaluronan
HtrA: High-temperature requirement A
RHAMM: Hyaluronan-mediated motility receptor
HIF-1α: Hypoxia-inducible factor 1α
HRE: Hypoxia response element
IGF-1R: Insulin-like growth factor receptor 1
ITG: Integrin
ICAM-1: Intercellular adhesion molecule 1
IL: Interleukin
ILVs: Intraluminal vesicles
KLRG1: Killer cell lectin-like receptor G1
KLF: Kruppel-like factor
L1CAM: L1 cell adhesion molecule
LGL: Lethal giant larvae
LC3-II: Light chain 3-II
LGSOC: Low-grade serous ovarian cancer
LFA: Lymphocyte function-associated molecule-1
MHC: Major histocompatibility complex
MMP: Matrix metalloproteinase
MET: Mesenchymal-to-epithelial transition
MMT: Mesothelial–mesenchymal transition
MSI: Microsatellite instability
MAPK/ERK: Mitogen-activated protein kinase/extracellular signal-regulated kinase pathway
MVB: Multivesicular bodies
NECL: Nectin-like family
N-cadherin: Neural cadherin
nSMase2: Neutral sphingomyelinase 2
NOX: Nicotinamide adenine dinucleotide phosphate oxidase
NNMT: Nicotinamide *N*-methyltransferase
ncRNA: Non-coding RNA
OCT4: Octamer-binding transcription factor 4
PATJ: PALS1-associated tight-junction protein
PDAC: Pancreatic ductal adenocarcinoma
PAR: Partitioning defective
PMCs: Peritoneal mesothelial cells
PM: Peritoneal metastasis
PTEN: Phosphatase and tensin homolog
PI3K/Akt/mTOR: Phosphatidylinositol 3-kinase/Akt/mammalian target of rapamycin
PDGFR: Platelet-derived growth factor receptor
PECAM-1: Platelet–endothelial cell adhesion molecule 1
PD-L1: Programmed death-ligand 1
PALS1: Protein associated with Lin-7 1
PHD3: Prolyl 4-hydroxylase 3
Rab: Ras-associated binding protein
G3BP1: Ras-GTPase-activating protein-binding protein 1
ROCK: RHO-associated protein kinase
SCRIB: Scribble polarity complexes
SLEX: Sialyl-LewisX
SNHG12: Small nucleolar RNA host gene 12
SNAIL: Snail family zinc finger transcription factors
SNARE: Soluble *N*-ethylmaleimide-sensitive factor attachment protein receptor
SPOCD1: Spen paralogue and orthologue C-terminal domain containing 1
SDF-1: Stromal cell-derived factor-1
SMAD7: Suppressor of mothers against decapentaplegic 7
TIM-3: T-cell immunoglobulin and mucin domain-containing protein 3
TCGA: The Cancer Genome Atlas
TIMP-2: Tissue inhibitor of metalloproteinases 2
TLR-4: Toll-like receptor-4
TGF: Transforming growth factor
TME: Tumour microenvironment
TP53: Tumour protein 53
TSAP6: Tumour suppressor-activated pathway 6
TSG101: Tumour susceptibility gene 101
TWISTs: Twist family bHLH transcription factors
VCAM-1: Vascular cell adhesion molecule 1
VEGF: Vascular endothelial growth factor
VISTA: V-domain Ig suppressor of T cell activation
ZEBs: Zinc finger E-box binding homeoboxes
ZO-1: Zonula occludens tight junction protein
